# Synthesis and Biological Evaluation of 3-Amidoquinuclidine Quaternary Ammonium Compounds as New Soft Antibacterial Agents

**DOI:** 10.3390/ph16020187

**Published:** 2023-01-25

**Authors:** Renata Odžak, Doris Crnčević, Antonio Sabljić, Ines Primožič, Matilda Šprung

**Affiliations:** 1 Department of Chemistry, Faculty of Science, University of Split, R. Bošković 33, 21000 Split, Croatia; 2 Doctoral Study of Biophysics, Faculty of Science, University of Split, R. Bošković 33, 21000 Split, Croatia; 3 Department of Chemistry, Faculty of Science, University of Zagreb, Horvatovac 102a, 10000 Zagreb, Croatia

**Keywords:** soft antimicrobial agents, quaternary ammonium salts, quinuclidine scaffold

## Abstract

Quaternary ammonium compounds (QACs) are among the most effective antimicrobial agents that have been used for more than a century. However, due to the growing trend of bacterial resistance and high toxicity of QACs, research in this field remains a pressing matter. Recent studies of the structure–activity relationship suggest that the introduction of the amide functional group into QAC structures results in soft variants that retain their antimicrobial properties while opening the possibility of fine-tuned activity regulation. Here, we report the synthesis and structure-function study of three structurally distinct series of naturally derived soft QACs. The obtained 3-amidoquinuclidine QACs showed a broad range of antibacterial activities related to the hydrophobic-hydrophilic balance of the QAC structures. All three series yielded candidates with minimal inhibitory concentrations (MIC) in the single-digit μM range. Time-resolved growth analysis revealed subtle differences in the antibacterial activity of the selected candidates. The versatile MIC values were recorded in different nutrient media, suggesting that the media composition may have a dramatic impact on the antibacterial potential. The new QACs were found to have excellent potential to suppress bacterial biofilm formation while exhibiting low ability to induce bacterial resistance. In addition, the selected candidates were found to be less toxic than commercially available QACs and proved to be potential substrates for protease degradation. These data suggest that 3-amidoquinuclidine QACs could be considered as novel antimicrobial agents that pose a low threat to ecosystems and human health.

## 1. Introduction

Membrane-acting cationic amphiphiles, such are quaternary ammonium compounds (QACs), are among the most potent antimicrobial agents with a broad spectrum of activity against bacteria, fungi, and certain viruses [1,2]. Their antibacterial mode of action is based on the electrostatic interaction between negatively charged groups on the bacterial cell surface and the positive charge of the quaternized center, leading to the subsequent penetration of an alkyl chain, membrane perforation, and cell death [3]. As indispensable components of many disinfectants and antiseptics, QACs are used for surface sterilization to combat and control the spread of infectious diseases in various environments. In addition, they serve as vehicles for drug delivery, vectors for gene transfection, biological dyes, and are often ingredients in skin and personal care products [2,4]. The widespread use of these compounds makes them high production volume chemicals, with over one million pounds produced or imported annually [5].

Such overuse of QACs, in addition to their specific physicochemical properties, leads to their accumulation in the environment. This is of concern for several reasons. First, due to their strong affinity for organic and inorganic particles in soil, a large fraction of QACs from surface waters are retained in soil at subinhibitory concentrations, promoting the development of bacterial resistance, which has increased at an alarming rate in recent years [5,6]. In addition, these compounds have been shown to be toxic to mammalian cells, and exposure to QACs can cause skin irritation and promote the development of asthma and inflammation, as well as induce genetic alterations [6].

To date, several research groups have successfully produced so-called soft QAC variants that retain their potent antimicrobial activity but can be readily hydrolyzed to yield inactive, nontoxic products [4,7,8,9,10]. In order for these compounds to be suitable for biomedical applications, they must be sufficiently stable under storage and sterilization conditions and non-toxic to mammalian cells. The first prepared soft QACs were esterquats [11], then amidequats as mono- and bis-QACs variants with different core structures [8,9,10]. Esterquats have been shown to be more susceptible to spontaneous or enzymatic biodegradation compared to amidequats, resulting in inactivation before they reach their optimal antimicrobial activity [8,12]. Moreover, in some cases, variants of esterquats have been found to be more toxic to human cells than to bacteria [8], leading to the adaptation of the non-natural peptidomimetic approach [4,13], i.e., incorporation of a more stable but less toxic amide functional group into the structures of QACs.

Based on these findings and our previous investigations on the bicyclic scaffold of quinuclidine [14,15], we endeavor to further explore this natural compound by peptidomimetic functionalization to obtain naturally derived soft QAC variants.

Previously published studies analyzing the structure–activity relationship of QACs have mainly investigated the polar to nonpolar ratio in the molecule, the effect of different counterions, the number of charged groups and their separation, polymeric scaffolds, and the scaffold structure as well as the influence of structural rigidity [16,17]. Although the effects of the three-dimensional structure of QACs on antimicrobial activity and toxicity have been observed, it is still unclear whether the structural rigidity due to sp^2^ hybridization or the polarity of the functional group on the scaffold is more influential in amide and ester bond-containing soft derivatives.

Recently, our group has shown that the addition of a polar oxime group to the heterocyclic core structure of pyridine-derived QACs impairs antimicrobial activity compared to the alkyl analog cetylpyridinium chloride (CPC) [18]. However, to our knowledge, there are no reports on how the addition of a polar group, especially a polar group on the cyclic core of QACs scaffolds, affects biological activity. With the aim to prepare soft QACs variants, we report here the synthesis of 3-amidoquinuclidine QACs and the investigation of how the addition of a polar amide group affects the antimicrobial activity of the corresponding QACs. We speculated that the addition of amide functionality would result in soft QACs with comparable antimicrobial activity to the corresponding alkyl analogs while allowing relatively easy enzymatic degradation. Therefore, the antimicrobial activity of the synthesized QACs was investigated against bacteria suspended in various media, as well as in the sessile form of the biofilm. In addition, the potential for the development of bacterial resistance of the candidate compound as well as its potential to serve as a substrate for degradation by trypsin protease were also investigated in this study.

## 2. Results and Discussion

### 2.1. Synthesis

**Scheme 1:**
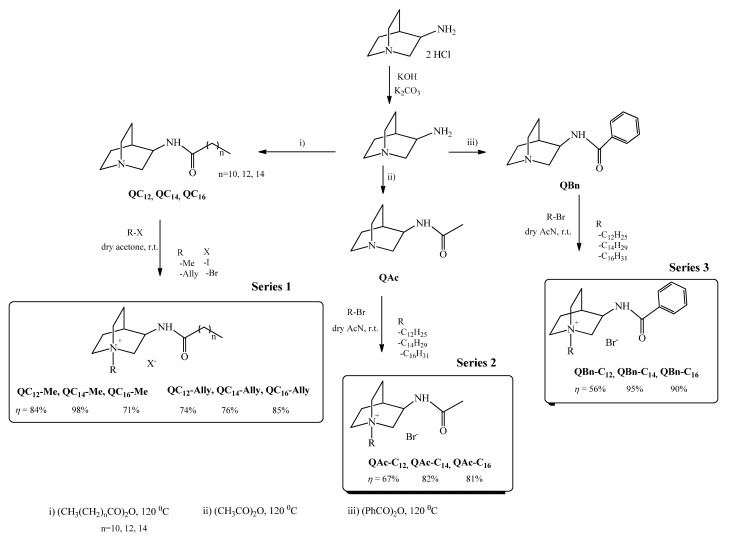
Synthetic pathway of three different 3-amidoquinuclidine precursors and their quaternary ammonium compounds (QACs). Reaction conditions for precursors synthesis were as follows: (**i**) (CH_3_(CH_2_)_n_CO)_2_O, *n* = 10, 12, 14; (**ii**) (CH_3_CO)_2_O; (**iii**) (C_6_H_5_CO)_2_O at 120 °C.

A quinuclidine scaffold containing a hydrolysis-prone amide bond served as the basis for the preparation of the novel soft quaternary ammonium compounds (QACs). Therefore, the first synthetic step was to prepare precursors of different 3-amidoquinuclidines (Scheme 1). A nucleophilic acyl substitution between 3-aminoquinuclidine and the corresponding anhydride in excess was carried out in the absence of a solvent at 120 °C. The series **1** precursors (**QC_12_**, **QC_14_**, **QC_16_**), with the desired purity, were obtained in good yields (70–98%), while the synthesis of the other two precursors, namely **QAc** and **QBn**, which served as starting materials for series **2** and **3**, has been described previously [19,20]. The obtained precursors were further quaternized to obtain three structurally distinct series of soft QACs to investigate the influence of the polar amide group and the positioning of the long chain on the antibacterial activity.

As we previously reported, the presence of polar groups in the QAC structures can significantly alter the antibacterial activity [18]. When comparing the structures of the soft QAC series, two main structural features should be considered. The first is the placement of the alkyl chain, and the second is the hydrophobicity of the group adjacent to the amide bond. Series **1** QACs have alkyl chains in the extension of the amide functionality, as opposed to series **2** and **3**, which have long chains at the tertiary nitrogen center of the 3-amidoquinuclidine backbone. In addition, long hydrophobic chains located near the amide bond, as in the series **1** QACs, are likely to sufficiently compensate for the polar amide nature, which is not the case for series **2**. For this reason, the series **3** QACs were synthesized, which contain a hydrophobic benzene moiety next to the amide group and long alkyl chains at the tertiary nitrogen atom.

Series **1** QACs were obtained in good yields (71–98%), but repeated crystallization was required to achieve the appropriate purity of the compounds (Scheme 1). Due to the bulkiness and strong hydrophobicity of precursors **QC_12_**, **QC_14_**, and **QC_16_**, the quaternization reagents were only one and three carbon atoms long. In contrast, the series **2** and **3** precursors, **QAc** and **QBn**, were quaternized with alkyl reagents with a chain length from 12 to 16 carbon atoms (Scheme 1). All QACs of series **2** and **3** were obtained in good yields of 67–81% and 56–95%, respectively, and were repeatedly crystallized to achieve the appropriate purity. After all new compounds were synthesized and characterized (Supplementary Material S1), we set out to investigate their antimicrobial potential.

### 2.2. Antimicrobial Activity

The antimicrobial activity of the synthesized precursors and their corresponding amidoquinuclidine salts was investigated by determining the minimum inhibitory concentrations (MICs). In addition to the newly synthesized compounds, two commercial QACs, cetylpyridinium chloride (CPC) and benzalkonium bromide (benzyldimethyldodecylammonium bromide, BAB) were included in the screening assays for comparison. MICs were determined for six Gram-positive strains, namely *Staphylococcus aureus* (ATCC25923; methicillin-resistant, MRSA; and ATCC 33591); *Bacillus cereus* (ATCC 14579); *Listeria monocytogenes* (ATCC 7644) and *Enterococcus faecalis* (ATCC 29212); and three Gram-negative strains, *Escherichia coli* (ATCC 25922), *Salmonella enterica* (food isolate), and *Pseudomonas aeruginosa* (ATCC 27853).

In general, all 3-amidoquinuclidine precursors lacked antibacterial activity (MICs ≥100 μM) (Supplementary Material S2), with the exception of the series **1** precursors, particularly **QC_14_**, which was good to moderately active against all Gram-positive bacteria, and **QC_16_**, which was moderately active against *S. aureus* ATCC 25923. This was expected because the series **1** precursors contain the alkyl chain in the extension of the amide bond, which induces perforation of the bacterial membrane. However, the extent of chain penetration into the membrane for these precursors and their quaternary salts might be limited by the planarity of the amide bond, which prevents free rotation of the N-C bond and projection of the alkyl chains in the membrane.

In contrast to the precursors, the quaternary 3-amidoquinuclidine compounds displayed a broad spectrum of antibacterial activities against both Gram-negative and Gram-positive bacteria.

As expected, the activities against Gram-negative strains (Table 1) were lower, which is consistent with the generally established observation that QACs exhibit lower activity against Gram-negative bacteria due to membrane-related characteristics [2]. This was also the case in our study, where the activities of the synthesized QACs were as low as those of the commercial CPC and BAB. Whereas all amidoquinuclidine QACs were practically inactive against Gram-negative strains, only benzylated derivatives showed somewhat moderate activity with **QBn-C_14_** and **QBn-C_16_**, being moderately active against *E. coli* and *S. enterica*. In addition, **QBn-C_14_** showed activity against *P. aeruginosa*, albeit at a high 63 μM.

On the other side, activity against Gram-positive strains was a bit puzzling. Although previous reports found that QACs functionalized with the amide bond in an alkyl chain have better antibacterial activity than their analogs without amide functionality, this was not the case in our study [16]. As shown in Table 1, MIC values ranged from single-digit values to values greater than 100 μM. Since the series 1 precursors, namely **QC_14_** and **QC_16_**, were found to be active, we hoped that their quaternization would further enhance their antibacterial properties. Indeed, quaternization of these precursors with methyl or allyl groups (**QC_14_-Me**, **QC_14_-Ally**, **QC_16_-Me**, **QC_16_-Ally**) improved their MICs, whereas in some cases, e.g., *L. monocytogenes*, the MIC was reduced to single-digit values. Since methylated and allylated QACs exhibited similar antibacterial potential, it led us to the conclusion that positive nitrogen center and long alkyl chains are indeed two indispensable structural features of QACs.

Since the antibacterial activity of series **1** showed a diverse range of MIC values and did not meet the desired expectations, we designed and synthesized two other series of 3-amidoquinuclidine salts to further investigate the structure–activity relationship in relation to the presence of the amide bond. In designing the other two series of 3-amidoquinuclidine QACs, we considered the following structural elements. First, we considered the positioning of the chain and, second, the polarity of the amide group on the quinuclidine scaffold.

To investigate the effects of chain position and amide group on the bioactivity of quinuclidine, the acetamide series of QACs was synthesized. In this series, the amide functionality was retained at the 3C atom of the core but with long alkyl chains extending from the quaternary center. The poor performance of the acetamides came as a surprise given that these derivatives have a plain alkyl chain extending from the quaternary center (Table 1). Indeed, in series 1, the chain at the 3C atom of the quinuclidine backbone is additionally functionalized with an amide group. Considering the planarity of the amide bond, it can thus be assumed that the series **1** chain penetrates the bacterial membrane only to a limited extent. For the acetamide series, we assumed that the intercalation of the chain into the lipid bilayer would not be disrupted by the presence of the amide bond; therefore, lower MIC values were expected. However, this was not the case, suggesting that the incorporation of a polar group into the cyclic core impairs antibacterial activity even when the plain alkyl chain is located at the quaternary center. This was observed in our previous study with a structural analog of CPC differing only in the presence of an oxime group on the heterocyclic pyridinium core. The resulting QACs were practically inactive due to the strong negative influence of the polar oxime group on the otherwise highly bioactive structure of CPC [18].

To further investigate this hypothesis, we additionally synthesized benzylated series of 3-amidoquinuclidine with long alkyl chains at the tertiary center on the assumption that the addition of the benzyl ring would increase the lipophilicity of the quinuclidine core. As shown in Table 1, the addition of the benzyl ring in the extension of amide functional group resulted in **QBn-C_12_** with observed MICs of 31 and 63 μM and **QBn-C_14_** and **QBn-C_16_** with single-digit MIC values against *L. monocytogenes* and *E. faecalis*, respectively.

The striking feature in all three series was the excellent activity against *L. monocytogenes* at low micromolar values (4 and 8 μM), suggesting that the 3-amidoquinuclidine salts may have a mechanism of action specific to this bacterium in addition to their effect on the membrane. This is particularly interesting considering that a recent study highlighted the occurrence of QACs tolerance in *L. monocytogenes* [21], so further studies on the mode of action of 3-amidoquinuclidine QACs against this pathogen are currently underway in our laboratory.

In summary, the addition of polar groups to otherwise nonpolar scaffolds in QACs structures is a structural feature that is not well tolerated, as it leads to derivatives with lower biological potential. Reducing the polarity of such structures by adding hydrophobic groups could restore antibacterial efficacy by allowing easier anchoring and further incorporation into the bacterial membrane. The only structural requirement for antimicrobial activity of monoQACs is a finely tuned hydrophilic-hydrophobic character of the core structure in addition to the length of the alkyl chain. These results represent a new addition to the already-known structural feature of hydrophilic-hydrophobic balance in amphiphilic compounds responsible for antibacterial activity [1], and our results prove that a fine-tuned modulation of this balance can strongly influence the bioactivity of such compounds.

### 2.3. The Influence of the New 3-Amidoquinuclidine QACs on Growth Dynamics of Staphylococcus aureus ATCC 25923

The presence of an antibacterial agent can specifically alter and influence the dynamics of bacterial growth, resolving a subtle difference between the antibacterial agents tested. Therefore, in addition to endpoint susceptibility tests, such as the microdilution method to determine the minimum inhibitory concentration, time-resolved growth analysis provides more detailed information about bacterial growth at temporal resolution in the presence of antibiotics [22].

**Figure 1:**
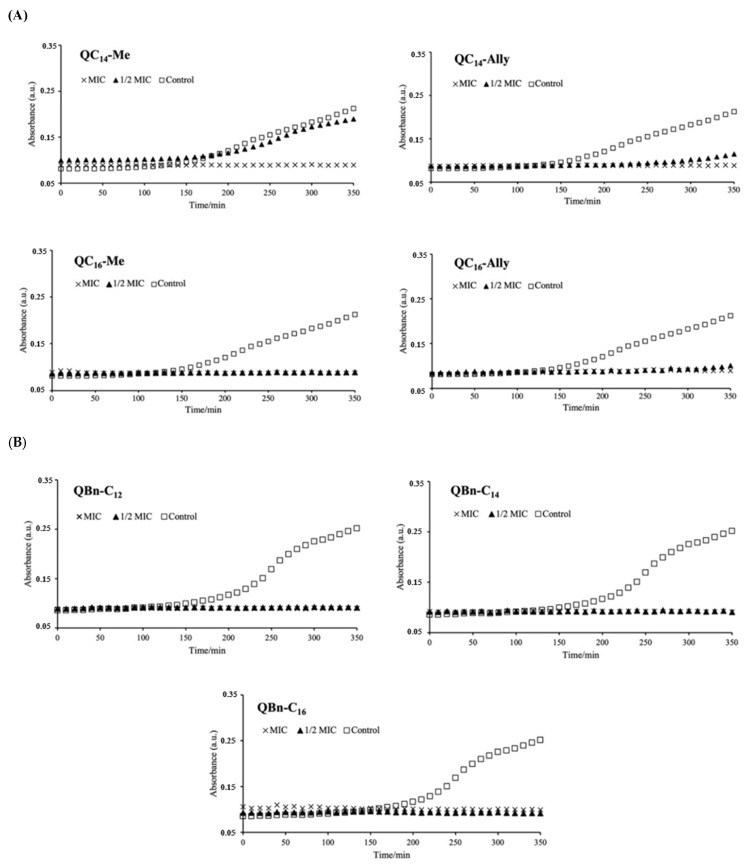
Time-resolved growth analysis of *Staphylococcus aureus* ATCC 25923 during ~6 h incubation with no antibacterial agent (control) and at MIC and ½ MIC of series **1 QC_14_-Me**, **QC_14_-Ally**, **QC_16_-Me**, and **QC_16_-Ally** (**A**) and series **3 QBn-C_12_**, **QBn-C_14_**, and **QBn-C_16_** (**B**).

The growth kinetic experiments were performed to further investigate the efficacy of the selected 3-amidoquinnuclidine candidates. The candidates from series **1** (**QC_14_-Me**, **QC_14_-Ally**, **QC_16_-Me**, and **QC_16_-Ally**) and series **3** (**QBn-C_12_**, **QBn-C_14_**, and **QBn-C_16_**) were incubated at MIC and ½ MIC for 350 min with an appropriate number of CFU of *Staphylococcus aureus* ATCC 25923 at 37 °C with shaking. Time-resolved growth analysis was performed along with the control, in which bacterial growth was monitored without the presence of the antibacterial agent. The resulting time-resolved growth curves in Figure 1A,B show that not all candidates suppressed bacterial growth to the same extent.

For example, **QC_14_-Me** and **QC_14_-Ally** were unable to inhibit bacterial growth at concentrations half the corresponding MICs, while the other candidates in series **1** and **3** effectively suppressed bacterial growth within the experimental period of ~6 h. The ability of QACs to inhibit bacterial growth at concentrations below the MIC has already been demonstrated in our studies with benzylimidazole- and quinuclidinol-derived QACs [14,23]. In these studies, some antimicrobial candidates showed suppression of bacterial growth even at ¼ of the MIC, suggesting better antibacterial efficacy of these candidates.

The differences in the antibacterial efficacy of **QC_14_-Me** and **QC_14_-Ally** became apparent when their growth curves were examined in more detail. As we approach the end of the growth curves, a slight difference in the suppression of bacterial growth between these candidates becomes more apparent. Compared to **QC_14_-Me**, **QC_14_-Ally** shows prolonged growth inhibition, indicating a subtle difference in the antibacterial efficacy of these two candidates.

The time-resolved growth analysis and MIC data suggest that the identified series **1** and **3** candidates indeed show promising antibacterial potential.

### 2.4. Antimicrobial Activity in Different Nutrient Media

**Figure 2:**
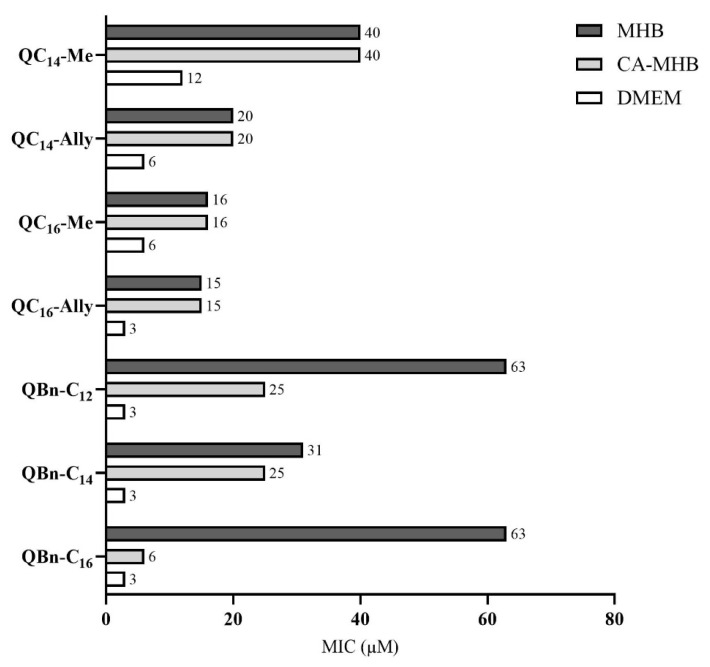
Minimal inhibitory concentrations (μM) in Mueller–Hinton Broth (MHB), cation-adjusted MHB, and Dulbecco’s Modified Eagle Medium (DMEM) for selected candidate quaternary ammonium compounds against *Staphylococcus aureus* ATCC 25923.

It has been shown that the nature of the culture media can have a significant effect on antibacterial activity, especially on the antibacterial activity of small cationic molecules such as quaternary ammonium compounds [13]. The main reason for this is the presence of anions in the nutrient media, which may affect the electrostatic interaction of small cationic amphiphiles with the bacterial membrane. Therefore, we investigated the influence of the culture media on the antibacterial activity of selected candidates against *Staphylococcus aureus* ATCC 25923 (Figure 2). Antibacterial activity was determined in two different media, cation-adjusted MHB (CA-MHB) and simple Dulbecco’s Modified Eagle Medium (DMEM).

The main component of Mueller–Hinton broth (MHB) is casein hydrolysate, which contains negatively charged amino acids and small peptides that could hinder the electrostatic interaction between the positive nitrogen of QAC and the negative groups on the bacterial membrane.

Cation-adjusted MBH (CA-MHB) contains calcium and magnesium ions and is regularly used for the susceptibility testing of Gram-positive and Gram-negative bacteria against different antimicrobial agents. In this study, we used CA-MHB under the assumption that the tested QACs in this medium compete with divalent metal cations for negative groups (e.g., teichoic acid) at the bacterial cell surface and mimic the excess of positive charge as bisQACs; therefore, we expected lower MIC values. As can be seen in Figure 2, the MIC values of the benzylated candidates actually decreased between one and tenfold, with the largest decrease in MIC observed for **QBn-C_16_**. However, the series **1** candidates had the same MIC values in both MHB versions. The explanation for this could be the position of the alkyl chain, which, in series **1**, is located in the extension of the sp^2^-hybridized carbon, which could hinder the rotation of the chain and its placement in the lipid bilayer. This has also been noted in studies investigating the relationship between rigidity and activity of QACs, in which the authors speculated about a specific projection and restricted rotation of the aliphatic side chains due to the sp^2^-hybridized carbon, leading to lower bioactivity in esterquats [8,16,17].

To further investigate the effects of nutrient media on MIC, MIC values were determined in relatively simple Dulbecco’s Modified Eagle Medium (DMEM) containing glucose as the main carbon source for bacterial growth, glutamine, sodium pyruvate, lipoic acid, vitamin B_12_, biotin, ascorbic acid, and phenol red. In contrast to the MICs obtained in CA-MHB, lower MICs were observed in DMEM for all our candidates. More specifically, all our candidates showed excellent antibacterial activity in DMEM with MIC values ranging from 3 to 12 μM, lowering the initial MIC by up to 21-fold. One explanation for this could be the absence of the anionic peptides and amino acids in the culture media (CA-MHB and DMEM) and/or the synergistic effect of our candidates with other antimicrobial agents present in DMEM (e.g., ascorbic acid and lipoic acid) [24].

From these results, we can conclude that the components of the culture medium play a crucial role in determining antibacterial efficacy, especially those of the cationic small molecules, as they can hinder the otherwise strong interaction with the bacterial membrane, which is probably the first step of the antibacterial mode of action.

### 2.5. Inhibition of the Biofilm Formation of Staphylococcus aureus ATCC 25923

Bacterial biofilms are complex multicellular communities of Gram-positive or Gram-negative bacteria held together by an extracellular polymeric matrix. In this form, the bacteria are usually resistant to antibacterial agents and therefore pose a major threat to human health by invading and colonizing medical devices [13,25]. One of the most common bacteria causing nosocomial infections in humans is *Staphylococcus aureus*, especially MRSA strains, which are resistant to almost all available β-lactams and other classes of antibiotics [26,27]. It has been speculated that the presence of a permanent positive charge on cationic amphiphiles may facilitate penetration through the extracellular polymeric matrix and induce drug uptake [28]. We and others have previously shown that QACs can successfully inhibit *S. aureus* biofilm formation at concentrations below the MIC [13,14,29].

**Figure 3:**
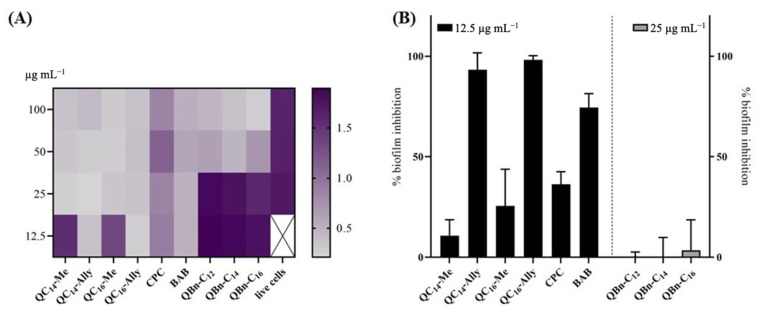
Inhibition of the *Staphylococcus aureus* ATCC 25923 biofilm upon treatment with candidate 3-amidoquinuclidine QACs, cetylpiridinium chloride (CPC), and benzyldodecyldimethylammonium bromide (BAB). The crystal violet heat map (**A**) shows the color intensity for all compounds at concentrations of 12.5, 25, 50, and 100 μg mL^−1^. The percentage of biofilm inhibition (**B**) for all compounds at concentrations of 12.5 and 25 μg mL^−1^. The values obtained were compared with the untreated control and are shown as the mean of at least three independent experiments performed in triplicate.

Therefore, we investigated the antibiofilm activity of the new 3-amidoquinuclidine QACs against the biofilm-forming pathogen *S. aureus* ATCC 25923, which causes opportunistic infections of various tissues in humans. Antibiofilm activity was compared with that of commercial BAB and CPC, which served as QACs standards. Antibiofilm activity was evaluated at four different concentrations (12.5, 25, 50, and 100 μg mL^−1^), and as shown on the crystal violet heat map (CV) (Figure 3A), all compounds have the potential to inhibit biofilm formation, albeit at different concentrations.

In the series of newly synthesized 3-amidoquinuclidine QACs, series 3 had a weak ability to inhibit bacterial biofilms, with almost no activity detected at a concentration of 25 μg mL^−1^ (Figure 3B). However, it should be noted that at twice the concentration, we observed inhibition of over 60% (Supplementary Information S3). In contrast to series **3**, series **1** and commercial QACs show excellent potential to inhibit biofilms, particularly **QC_14_-Ally** and **QC_16_-Ally**, which showed nearly 100% inhibition at a concentration of 12.5 μg mL^−1^, which is better than what we observed for CPC or BAB (Figure 3). These concentrations for **QC_14_-Ally** and **QC_16_-Ally** correspond to their determined MIC values, highlighting the good antibacterial potential of these candidates, which is worth further investigation.

### 2.6. The Potential for Bacterial Resistance Development

One of the major challenges that has emerged in the field of quaternary ammonium compounds in recent decades is bacterial resistance to this class of antimicrobial agents [30]. Research has shown that resistance is mainly, but not exclusively, related to specific efflux pumps that recognize and expel QACs from bacterial cells before they can irreversibly damage the cell and lead to cell death [31]. To date, several QAC-specific efflux pumps have been identified, but research has mainly focused on QacA and QacB, which belong to the Major Facilitator Superfamily (MFS) and are driven by proton motive force rather than ATP hydrolysis [32]. The protonophore, carbonyl cyanide 3-chlorophenylhydrazone (CCCP), disrupts the transmembrane electrochemical gradient and proton motive force, thus reducing ATP production [33]. This property of the CCCP reagent can be exploited to investigate whether QACs are substrates of efflux pumps, providing insight into the potential for resistance development. CCCP has been shown to decrease efflux activity of bacteria resistant to carbapenems [33], and similarly, *Staphylococcus aureus* ATCC 33591 strains resistant to QACs [31,34].

**Figure 4:**
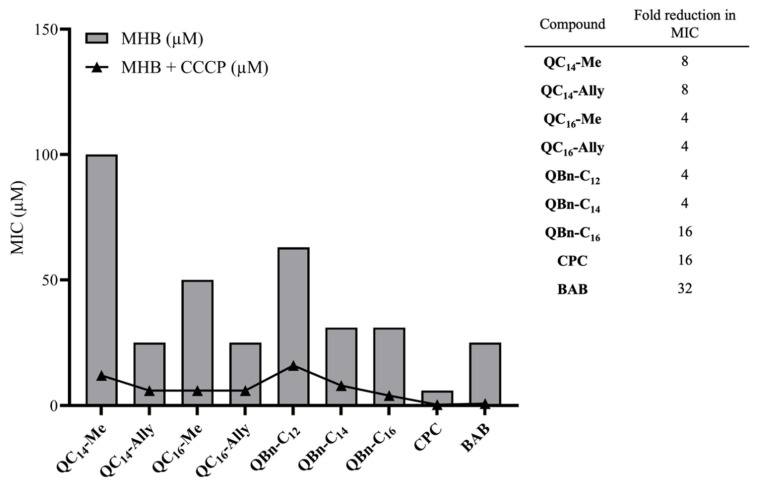
The potential for bacterial resistance development for 3-amidoquinuclidine quaternary ammonium compounds (QACs) and commercial QACs, cetylpiridinium chloride (CPC), and benzyldodecyldimethylammonium bromide (BAB). The obtained MIC values in Mueller–Hinton broth (MHB) are shown as bars in μM values compared to the MIC obtained in the presence of carbonyl cyanide 3-chlorophenylhydrazone (CCCP) (line). The bar graph is accompanied by a table showing the MIC reduction by the factor.

Therefore, considering the potential application of the identified 3-amidoquinuclidine QAC candidates, we further investigated their potential for bacterial resistance development. Minimum inhibitory concentrations were determined against *S. aureus* ATCC 33591 without or with the addition of CCCP (Figure 4). The results show that all tested QACs are substrates for efflux pumps as the MIC was decreased with CCCP (Figure 4). The greatest decrease in MIC was observed for the commercial QACs and **QBn-C_16_**, indicating that these compounds are the most favorable substrates for efflux pumps (table in Figure 4).

However, all other QACs prepared in this study showed a four- to eight-fold reduction in MIC, suggesting that newly synthesized QACs are not as favorable substrates for efflux as CPC and BAB. This may be explained by the regulation of the efflux pump expression. The Qac efflux pump expression is regulated by the transcription factor QacR. QacR has been shown to dissociate from the regulatory site after binding to QAC, allowing transcription on Qac efflux genes [35]. Comprehensive studies on QAC resistance conducted in the laboratories of Wuest and Minbiole have elucidated the role of QAC structure in resistance development. Namely, they found that multiQAC structures do not induce resistance, most likely due to their excessive positive charge, which limits their ability to accumulate in the cell and thus bind to QacR [36]. Considering that cellular accumulation of our 3-amidoquinuclidine QACs is similarly limited due to the polarity of the amide group on the quinuclidine scaffold, it is reasonable to assume that our candidates are also less prone to trigger bacterial resistance mediated by Qac efflux pumps despite the fact that our structures are monoQACs, which are known to be less effective than multiQACs structures.

### 2.7. Cytotoxicity of 3-Amidoquinuclidine Quaternary Ammonium Compounds

**Figure 5:**
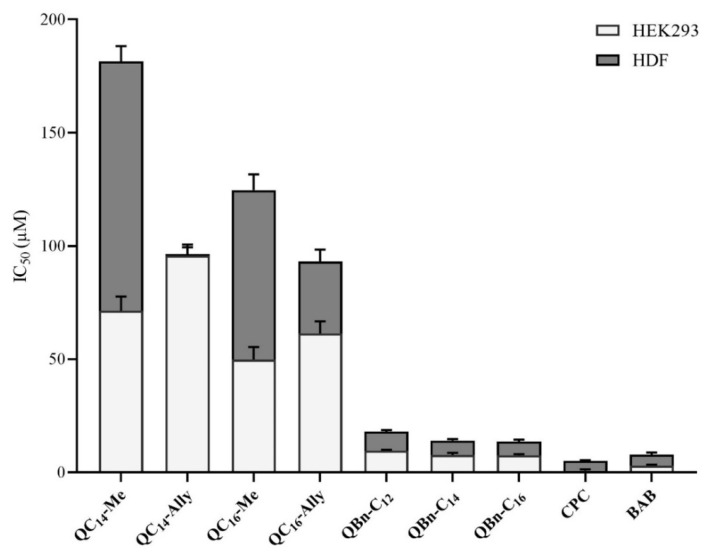
Cytotoxicity of 3-amidoquinuclidine quaternary ammonium compounds (QACs) and commercial QACs, cetylpiridinium chloride (CPC), and benzyldodecyldimethylammonium bromide (BAB). The IC_50_ values obtained are given as bars in μM values.

The cytotoxicity of 3-amidoquinuclidine QACs was determined using two human cell lines, namely human embryonic kidney (HEK293) and human dermal fibroblasts (HDF) (Figure 5). The determined IC_50_ values of the new and the commercial QACs indicate a much lower toxicity of the 3-amidoquinuclidine QACs. However, we must note that HEK293 appear to be more susceptible to the toxicity of QACs than HDF, which is expected because of the different cell line profile. Indeed, HEK293 are embryonic cells, which are inherently more sensitive than the differentiated, mature cells of human skin fibroblasts.

**Figure 6:**
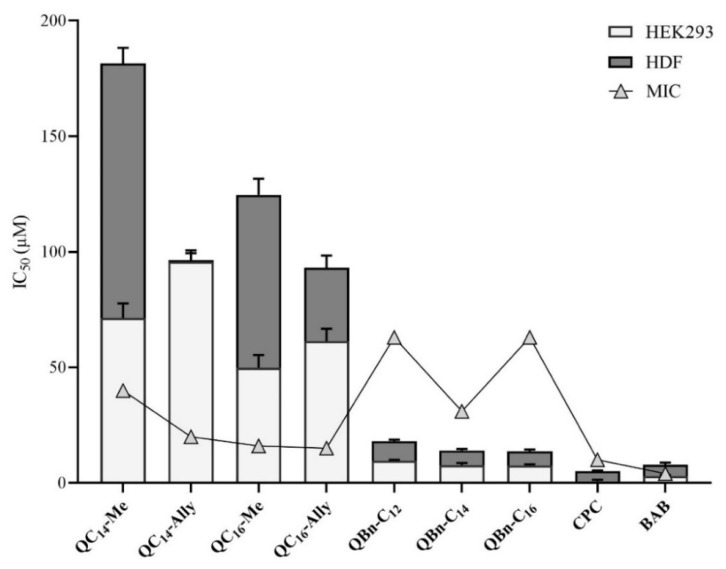
Cytotoxicity of 3-amidoquinuclidine quaternary ammonium compounds (QACs) and commercial QACs, cetylpiridinium chloride (CPC), and benzyldodecyldimethylammonium bromide (BAB). The IC_50_ values obtained are given as bars in μM values and compared to MIC against *Staphylococcus aureus* ATCC 25923 (line).

To further evaluate their biological efficacy, the IC_50_ values were compared with the minimum inhibitory concentrations determined for the candidate compounds against *Staphylococcus aureus* ATCC 25923 (Figure 6). A higher IC_50_ value compared to the MIC indicates better efficacy and safety of the compound.

As can be seen in Figure 6, the series **3** QACs had a higher MIC than IC_50_, indicating that these candidates are fundamentally toxic to human cells at concentrations lower than their corresponding MIC values. On the other hand, both cell lines showed excellent tolerance to all series **1** candidates, indicating that these compounds can be considered safe in the case of human exposure.

In conclusion, the cytotoxicity profile of selected 3-amidoquinuclidine QACs showed that the newly synthesized series **1** compounds exhibited low toxicity to human cell lines, which, together with their low MIC values, indicate that these compounds could be considered as potentially safe and promising new QAC candidates.

### 2.8. Protease Degradation of Amide Bond

**Figure 7:**
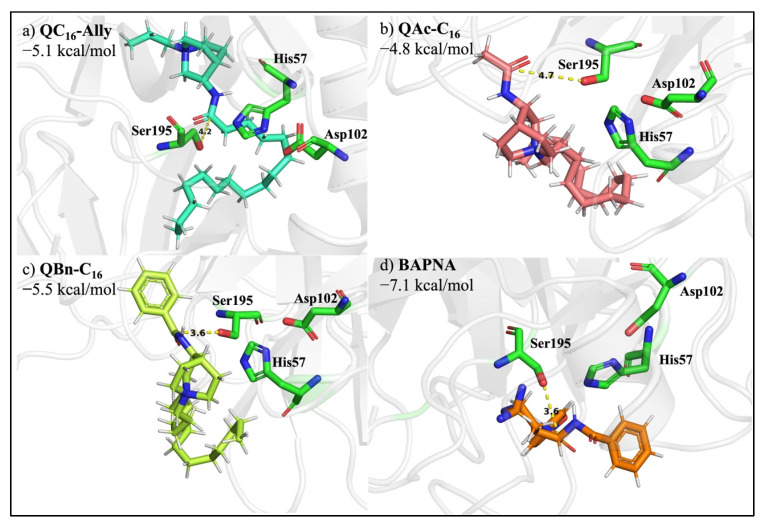
Docking analysis and binding affinities of **QC16-Ally**, (**a**) **QAc-C16** (**b**), and **QBn-C16** (**c**) in comparison with synthetic substrate BAPNA (**d**) as trypsin substrates. The snapshots illustrate the positioning of substrates within the enzyme active site with depicted catalytic amino acids, namely Ser195, Asp102, and His57.

The chemical degradation of an amide bond primarily implies its hydrolysis under specific extreme conditions, such as pH or temperature, while Kahne and Still found that the amide bond has a half-life of approximately 7 years when found in a water environment at neutral pH, proving that amides are the least reactive carboxylic acid derivatives [37]. The second and more common method of amide bond degradation is enzymatic digestion. Proteases are specific enzymes that are the primary driving force behind peptide bond degradation catalysis. Because of their potential to serve as specific drug targets, the pharmaceutical industry is paying increasing attention to this class of catalysts [38]. Therefore, we aimed to investigate the potency of our newly synthesized soft QACs to bind to the active site of the most abundant serine protease, trypsin. Representative QAC candidates of each synthesized series (**QC_16_-Ally**, **QAc-C_16_**, **QBn-C_16_**) were docked into the active site of trypsin to determine the binding affinity of each compound (Figure 7).

The DockingPie Vina plugin was used to execute the docking study in PyMOL, with the PDB-deposited trypsin structure 1AUJ [39]. Calculated binding affinities were compared with the binding affinity of Nα-benzoyl-DL-arginine-4-nitroanilide hydrochloride (BAPNA), a synthetic trypsin substrate. We discovered that each of the produced soft QACs function as a plausible trypsin-degradable substrate, with slight differences in their binding affinities. The binding affinity of **QBn-C_16_** (−5.5 kcal/mol) was most comparable to BAPNA’s (−7.1 kcal/mol), most likely because both compounds feature the benzene moiety as a structural component. Furthermore, although having slightly higher binding affinity values, **QAc-C_16_** and **QC_16_-Ally** also proved to act as suitable trypsin substrates. According to the estimated binding affinities of each QAC series representative compound, **QC_16_-Ally** stands out as the most noteworthy candidate to be pointed out, principally due to the long-chain positioning. Since the long chain is in extension of the amide bond further cleaved by trypsin, the enzymatic breakdown of such structure results in the biologically inactive degradation products. Consequently, due to the structural similarity, we can speculate that all series **1** QACs could be regarded as environmentally friendly because their degradation results in practically inactive products which are less likely to promote bacterial resistance or cause a toxic effect on ecosystems.

## 3. Materials and Methods

### 3.1. Synthesis

#### 3.1.1. Synthesis of (±)3-Aminoquinuclidine

A saturated solution of potassium hydroxide was added dropwise to commercially available (±)3-aminoquinuclidine dihydrochloride (Sigma Aldrich, Burlington, MA, USA) to pH > 7. The resulting mixture was agitated on the IDL MSH-20A magnetic stirrer followed by the chloroform extraction (10 × 5 mL). The collected chloroform layers were dried on anhydrous potassium carbonate. The solvent was evaporated on the Büchi rotary evaporator to provide (±)3-aminoquinuclidine, which served as a reactant to produce appropriate 3-amidoquinuclidine.

#### 3.1.2. Synthesis of (±)3-Amidoquinuclidine Precursors

For synthesis, (±)3-aminoquinuclidine and an excess of appropriate anhydrides of (lauric, myristic, and palmitic anhydride) were set to react on the magnetic stirrer in an oil bath at a temperature of 120 °C. The corresponding anhydrides are commercially available (Sigma Aldrich, Burlington, MA, USA) and were not further purified prior to reaction. Reaction progress was monitored by thin-layer chromatography using DC-Alufolien Aluminiumoxide 60 F_254_ plates (Merck, Rahway, NJ, USA) with 5:1 and 9:1 chloroform/methanol eluent. Upon complete consumption of starting primary amine, water was added, followed by sodium carbonate solution, until the pH reached ~11. The mixture was transferred to the separatory funnel and extracted with chloroform (10 × 10 mL). Chloroform layers were dried over anhydrous sodium sulfate. Solvent evaporation provided (±)3-amidoquinuclidine with long-chain (**QC_12_**, **QC_14_**, **QC_16_**) in very good yields (>80%). Then, (±)3-acetamidoquinuclidine (**QAc**) and (±)3-benzamidoquinuclidine (**QBn**) were synthesized as reported previously [19,20]. Finally, obtained different (±)3-amidoquinuclidines were further used as precursors for the synthesis of three series of QACs.

#### 3.1.3. Synthesis of Quaternary Ammonium Compounds

Series **1** QACs was prepared by the reaction of (±)3-amidoquinuclidines with a long alkyl chain and corresponding equimolar amount of quaternization reagents: methyl iodide and allyl bromide (Sigma Aldrich, Burlington, MA, USA). Reactions were carried out at room temperature in dry acetone with constant stirring over 2–3 days. Quaternization reactions for series **2** and **3** QACs were set up at room temperature in dry acetonitrile with constant stirring, along with an equimolar amount of appropriate quaternizing reagents: 1-bromododecane, 1-bromotetradecane, and 1-bromohexadecane (Alfa Aesar, Haverhill, MA, USA). Repeated crystallization was required to attain the desired purity of the compounds in each series.

### 3.2. Broth Microdilution Assays

#### 3.2.1. Minimum Inhibitory Concentration

The competence of newly synthesized (±)3-amidoquinuclidine quaternary ammonium compounds against bacteria was investigated on Gram-positive (*Staphylococcus aureus* ATCC 25923, *Staphylococcus aureus* ATCC 33591, *Bacillus cereus* ATCC 14579, *Listeria monocytogenes* ATCC 7644, *Enterococcus faecalis* ATCC 29212, and *Staphylococcus aureus* MRSA (clinical isolate)) and Gram-negative (*Escherichia coli* ATCC 25922, *Pseudomonas aeruginosa* ATCC 27853, and *Salmonella enterica* (food isolate)) bacterial strain representatives. American Type Culture Collection (ATCC) bacterial strains were purchased from BioGnost. The Broth Microdilution Assay was used to assess antibacterial activity in accordance with the Clinical and Laboratory Standard Institute’s Methods for Dilution Antimicrobial Susceptibility Test for Bacteria That Grow Aerobically [40]. The bacterial strain of interest was inoculated in fresh Mueller Hinton broth (MHB), (Biolife, Graz, Austria) using a sterile loop and cultivated overnight at 37 °C and continuous shaking at 220 rpm. The next day, the bacterial culture was diluted 10-fold in tempered MHB and propagated until the bacteria reached the exponential growth phase (OD_600_ = 0.3–0.5). Once the bacteria reached the preferential growth phase, the culture was diluted to 5 × 10^5^ CFU/mL. Using a multichannel pipette, 50 µL of the thus diluted cell culture was pipetted into each well of a 96-well plate containing twofold serial dilutions of the compounds in duplicates. After the bacterial culture was pipetted into each well, the 96-well plate contained compounds from 125 µM to 0.12 µM. The plates were incubated overnight at 37 °C. The minimum inhibitory concentration (MIC) was determined the next day as the lowest concentration of the compound that did not cause visible bacterial growth in the corresponding wells. Visual verification of the results was confirmed by the addition of 20 µL of 2-(4-iodophenyl)-3-(4-nitrophenyl)-5-phenyltetrazolium chloride (INT) reagent (6 mg/mL), which turns purple in the presence of viable bacterial cells.

#### 3.2.2. Minimum Inhibitory Concentration in Cation-Adjusted Mueller Hinton Broth (CA-MHB)

A representative bacterial strain, *Staphylococcus aureus* ATCC 25923, was grown overnight in Mueller Hinton broth (MHB). The following day, the culture was diluted in tempered MHB and allowed to grow at 37 °C with constant shaking of 220 rpm. Once the bacterial culture reached the exponential growth phase (OD_600_ = 0.3–0.5), it was diluted in the cation-adjusted Mueller Hinton broth (CA-MHB) containing MgCl_2_ (10 mM) to the final cell count of 5 × 10^5^ CFU/mL. The previously prepared 96-well plate contained twofold serial dilutions of the compounds to be tested, dissolved in CA-MHB, in the concentrations range of 125 µM to 0.12 µM. Then, 50 µL of the bacterial cells diluted in CA-MHB was pipetted in each well of the plate and incubated overnight at 37 °C with constant shaking at 220 rpm. The next day, the minimum inhibitory concentration (MIC) was determined as the lowest concentration of the compound that prevented supposed bacterial growth in the wells. Visually determined results of antibacterial activity were confirmed by the addition of 20 µL 2-(4-iodophenyl)-3-(4-nitrophenyl)-5-phenyltetrazolium chloride (INT) reagent (6 mg/mL). The MIC value of a drug in CA-MHB containing NaCl (85 mM) was determined using the same experimental technique.

#### 3.2.3. Minimum Inhibitory Concentration in Dulbecco’s Modified Eagle Medium (DMEM)

The overnight culture of *Staphylococcus aureus* ATCC 25923 was diluted in fresh Mueller Hinton broth (MHB) and placed in an orbital shaker incubator (Gallenkamp, Cambridge, UK) at 37 °C and 220 rpm. Once the culture reached the exponential growth phase (OD600 = 0.3–0.5), cells were centrifuged (Heraeus, Biofuge primo R, Hanau, Germany) at room temperature at a speed of 4000× *g*. The cell pellet was resuspended in tempered Dulbecco’s Modified Eagle Medium (DMEM) to a final concentration of 5 × 10^5^ CFU/mL. Then, 50 µL of as-prepared cells were added to a 96-well plate containing twofold serial dilutions of tested compounds in DMEM medium in the range of concentrations from 125 µM to 0.12 µM. The minimum inhibitory concentration (MIC) was visually assessed the next day. The MIC was determined as the lowest concentration of the substance at which no apparent bacterial growth was seen.

### 3.3. Time-Resolved Growth Analysis

Time-resolved growth analysis was performed in the microtiter plate reader (Bio-Tek EL808, Winooski, VT, USA) by incubating the selected antibacterial agent with *Staphylococcus aureus* ATCC 25923. The exponentially grown culture (OD_600_ = 0.3–0.5) of *S. aureus* ATCC 25923 was diluted to a final concentration of 5 × 10^4^ CFU/mL. Then, 50 µL of such prepared cell culture was added to the 96-well plates containing the MIC or ½ MIC of the compound to be tested. The plates were incubated at 37 °C for 350 min in the microplate reader with shaking. OD_600_ was recorded at 10 min intervals. The results obtained represent the mean of two independent experiments performed in triplicate.

### 3.4. Biofilm Inhibition Assay

Minimum biofilm inhibitory concentration (MBIC) was determined against *Staphylococcus aureus* ATCC 25923. Compounds were dissolved in MHB and serially diluted in 96-well plates with a final mass concentration ranging from 100 to 12.5 µg/mL. The exponentially grown culture (OD_600_ = 0.3–0.5) of *S. aureus* ATCC 25923 was diluted to a final concentration of 5 × 10^6^ CFU/mL. Then, 50 µL of such prepared cell culture was added to the 96-well plates containing twofold dilutions of the compound to be tested. The plates were incubated for 48 h. After incubation, the broth over the biofilm was aspirated, and the plate with the biofilm was dried at 60 °C. After drying, 100 µL of 1% Crystal Violet (CV) solution was pipetted into the wells and incubated for one hour at room temperature. Once the biofilm was stained, the CV solution was rinsed with Milli-Q water. The biofilm formed was also rinsed. Then, 100 µL of 70% ethanol was added to the wells to dissolve the CV stain. The plates were incubated for one hour, and the well content was resuspended with the multichannel pipette if necessary. Absorbance was measured at 595 nm in an optical reader. The percentage of biofilm formed was calculated by dividing the absorbance of the well containing the tested substance by the absorbance of the untreated bacterial cells and multiplying by 100.

### 3.5. Potential of Bacterial Resistance Development

Bacterial susceptibility toward (±)3-amidoquinuclidine quaternary ammonium compounds was evaluated in the presence of an ATP synthesis inhibitor carbonyl cyanide 3-chlorophenylhydrazone (CCCP) (Sigma Aldrich). The minimum inhibitory concentration (MIC) of CCCP against *Staphylococcus aureus* ATCC 33591 (MRSA) was established before the experiment. The overnight culture of *Staphylococcus aureus* ATCC 33591 was diluted in tempered Mueller Hinton broth (MHB) and cultivated in the orbital shaker at 37 °C and 220 rpm. When the culture reached the preexponential phase of growth (OD_600_ = 0.3–0.5), it was diluted to a final concentration of 5 × 10^5^ CFU/mL in MHB containing 10 µM CCCP. A total of 50 µL of prepared cell suspension was pipetted into a 96-well plate containing twofold serial dilutions of tested compounds in MHB with CCCP at a final concentration of 10 µM. Plates were incubated overnight, and the MIC reduction of tested compounds in the presence of 10 µM CCCP was recorded the following day.

### 3.6. Cytotoxicity

Three separate healthy human cell lines (HaCaT, HEK293, HDF) were used to investigate the cytotoxicity of the synthesized quaternary ammonium compounds. The results were compared to benzyldodecyldimethylammonium bromide (BAB), a commercially available quaternary ammonium salt. Human cells were cultured in DMEM medium at 37 °C in a humidified atmosphere with 5% CO_2_. Tested compounds were dissolved in DMEM medium and twofold serially diluted in a 96-well plate. Measurements were performed in duplicates in the range of compound concentrations from 250 µM to 0.25 µM. In each well of the plate, 5000 cells were seeded and incubated for 48 h. Then, 20 µL of MTS reagent was added to each well according to the manufacturer’s (CellTiter 96^®^ Aqueous One Solution Cell Proliferation Assay, Promega, Madison, WI, USA) instructions. Cells were incubated with the MTS reagent for an additional three hours followed by the absorbance measurement at 490 nm. IC_50_ values were obtained using GraFit 6.0 software by plotting the compound’s concentration versus absorbance.

### 3.7. Docking QC_16_-Ally to the Active Site of Serine Protease

A protein data bank (PDB) file of bovine trypsin complexed with a meta-cyano-benzylic inhibitor (1AUJ) served as the starting point for docking the selected compound to the most abundant serine peptidase protein [5]. After the PDB file was loaded into the PyMOL, the inhibitor and water molecules were removed, leaving the crystal structure of bovine trypsin ready for docking. After the ligands was imported into PyMOL as a mol.2 file, the sequence of 1AUJ was searched for the key amino acid residues of the active site. Both the protein and the ligand were added to the DockingPie 1.0.1. The Vina plugin console and the grid for docking were set with the appropriate coordinates (*x* = 24.98, *y* = 15.34, *z* = 20.88). The ligands were docked to the selected active site in 20 poses to obtain more accurate results of the binding energy.

## 4. Conclusions

Quaternary ammonium compounds (QACs) are among the most effective antimicrobial agents with the widespread use and application possibilities. The growing trend in bacterial resistance and high toxicity of QACs ensure continuous research in this field. Recent studies of the structure-activity relationship suggest that the introduction of the amide functional group into QAC structures results in soft variants that retain their antimicrobial properties while opening the possibility of fine-tuned activity regulation. Based on our previous findings that natural precursors can serve as scaffolds for new QACs development, here we report the synthesis and structure-function study of three structurally distinct series of naturally derived soft QACs. The obtained 3-amidoquinuclidine QACs showed a broad range of antibacterial activities that were correlated with the hydrophobic-hydrophilic balance of the structures. All three series yielded candidates with minimal inhibitory concentrations (MIC) in the single-digit μM range for specific bacteria, namely *Listeria monocytogenes*, which warrants further investigation. Time-resolved growth analysis revealed subtle differences in antibacterial activity among the selected candidates. The MIC values were determined in different nutrient media, suggesting that media composition can have a dramatic effect on antibacterial potential. The new QACs were found to have excellent potential to suppress bacterial biofilm formation while exhibiting low ability to induce bacterial resistance. In addition, the selected candidates were found to be less toxic than commercially available QACs and proved to be potential substrates for protease degradation. These data suggest that 3-amidoquinuclidine QACs could be considered as novel antimicrobial agents that pose a low threat to ecosystems and human health.

## Figures and Tables

**Table 1:** Minimal inhibitory concentrations (MICs) of 3-amidoquinuclidine quaternary ammonium compounds in μM.

Minimum Inhibitory Concentration (MIC)/µM										
***Compound***		**Gram-Positive**					**Gram-Negative**			
		*Staphylococcus aureus* ATCC25923	*Staphylococcus aureus* ATCC33591	*Staphylococcus aureus* Clinical/MRSA	*Bacillus cereus* ATCC14579	*Listeria monocytogenes* ATCC7644	*Enterococcus faecalis* ATCC29212	*Escherichia coli* ATTC25922	*Salmonella enterica* food isolate	*Pseudomonas aeruginosa* ATTC27853
***Series*** **1**	**QC_12_-Me**	100	>125	100	100	100	100	100	>125	>125
	**QC_12_-Ally**	>125	>125	>125	>125	>125	>125	>125	>125	>125
	**QC_14_-Me**	40	100	60	50	20	30	100	>125	>125
	**QC_14_-Ally**	20	50	25	25	**8**	15	50	>125	>125
	**QC_16_-Me**	16	25	31	>100	**8**	100	>100	>125	>125
	**QC_16_-Ally**	15	25	31	>100	16	>100	>100	>125	>125
***Series*** **2**	**QAc-C_12_**	>125	>125	>125	>125	>125	>125	>125	>125	>125
	**QAc-C_14_**	>125	>125	>125	>125	63	63	>125	>125	>125
	**QAc-C_16_**	>125	>125	>125	>125	**8**	31	>125	>125	>125
***Series*** **3**	**QBn-C_12_**	63	63	63	63	31	31	63	63	>125
	**QBn-C_14_**	31	31	31	63	**4**	**8**	31	31	63
	**QBn-C_16_**	63	31	31	63	**4**	**4**	31	63	>125
**Commercial** **QACs**	**CPC**	4	6	8	16	8	8	16	63	250
	**BAB**	10	25	25	13	10	15	63	50	>125

## Data Availability

Not applicable.

## Supplementary Materials

The following supporting information can be downloaded at: https://www.mdpi.com/article/10.3390/ph16020187/s1. Supplementary S1: ^1^H and ^13^C NMR spectra of the compounds, Table S1: Minimal inhibitory con-centration of QAC precursors, Figure S1: *Staphylococcus aureus* ATCC 25923 biofilm inhibition of the series **3** candidates at the compound concentration of 50 µg mL^−1^.
