# Supplementary material for: Synthesis and Biological Evaluation of 3-Amidoquinuclidine Quaternary Ammonium Compounds as New Soft Antibacterial Agents
Source: Pharmaceuticals (Basel). 2023 Jan 25;16(2):187. doi: 10.3390/ph16020187 (PMC9966435; doi:10.3390/ph16020187)
Supplement: Supplementary file 1 [file pharmaceuticals-16-00187-s001.zip › pharmaceuticals-2118086-supplementary.pdf]

Supplementary information

Supplementary Material S1

^{1}H NMR spectrum of **QC₁₂**

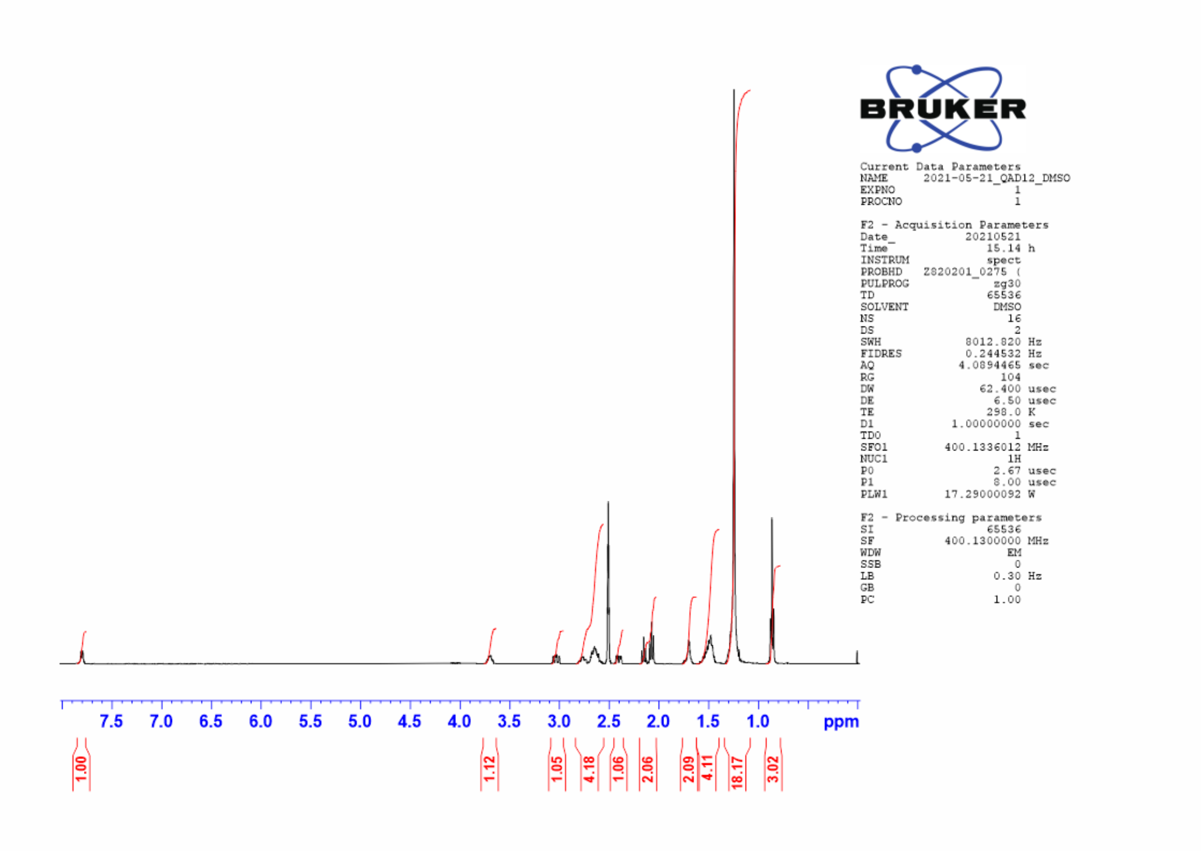

^{13}C NMR spectrum of **QC$_{12}$**

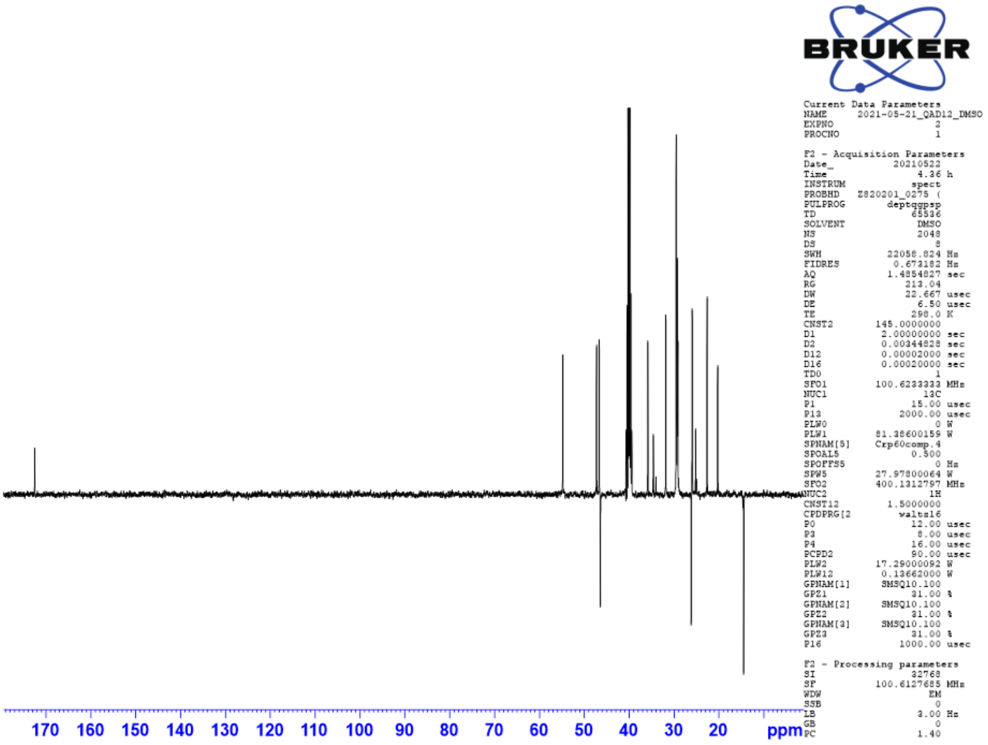

^{1}H NMR spectrum of **QC$_{14}$**

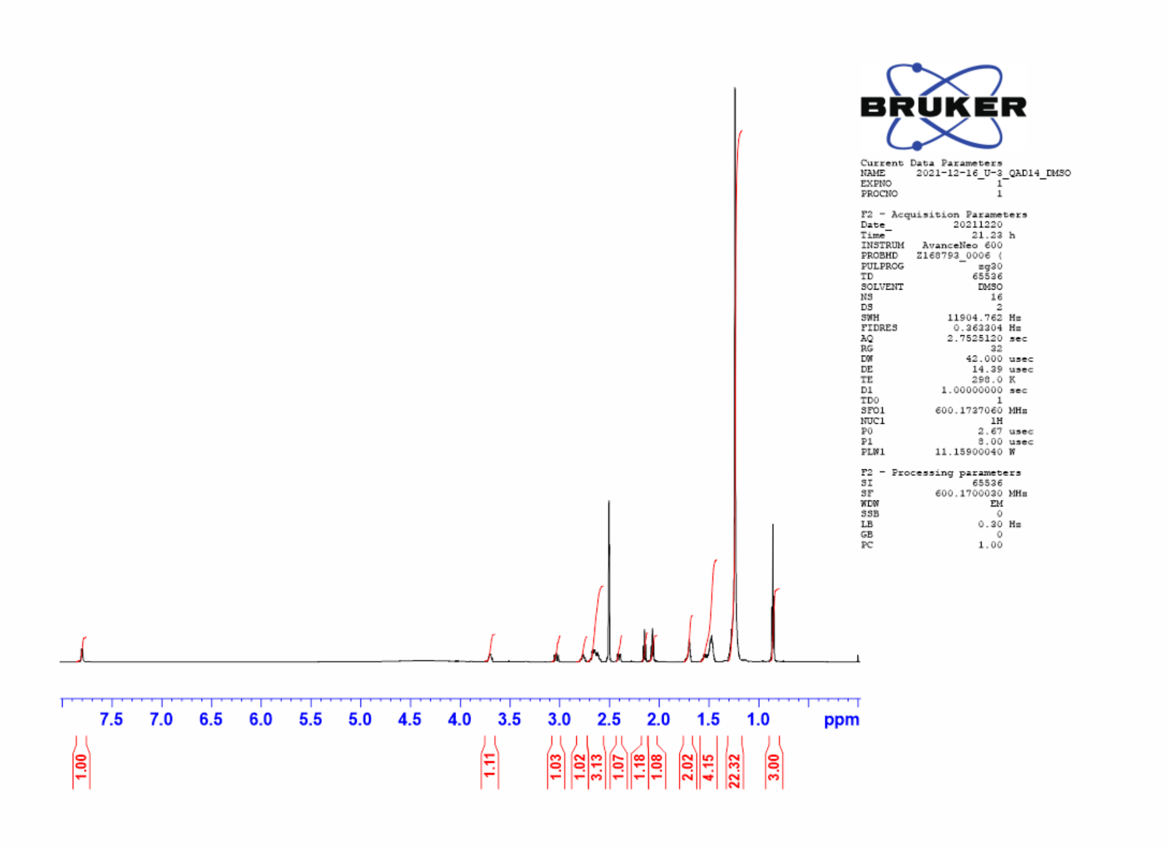

^{13}C NMR spectrum of **QC_{14}**

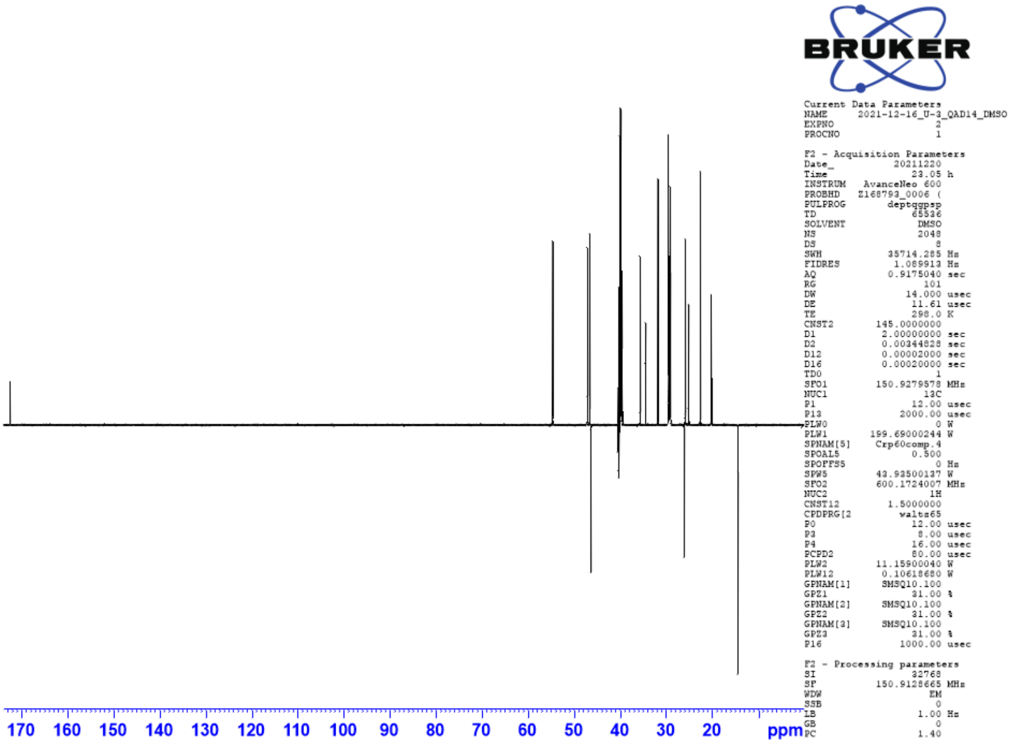

^{1}H NMR spectrum of **QC16**

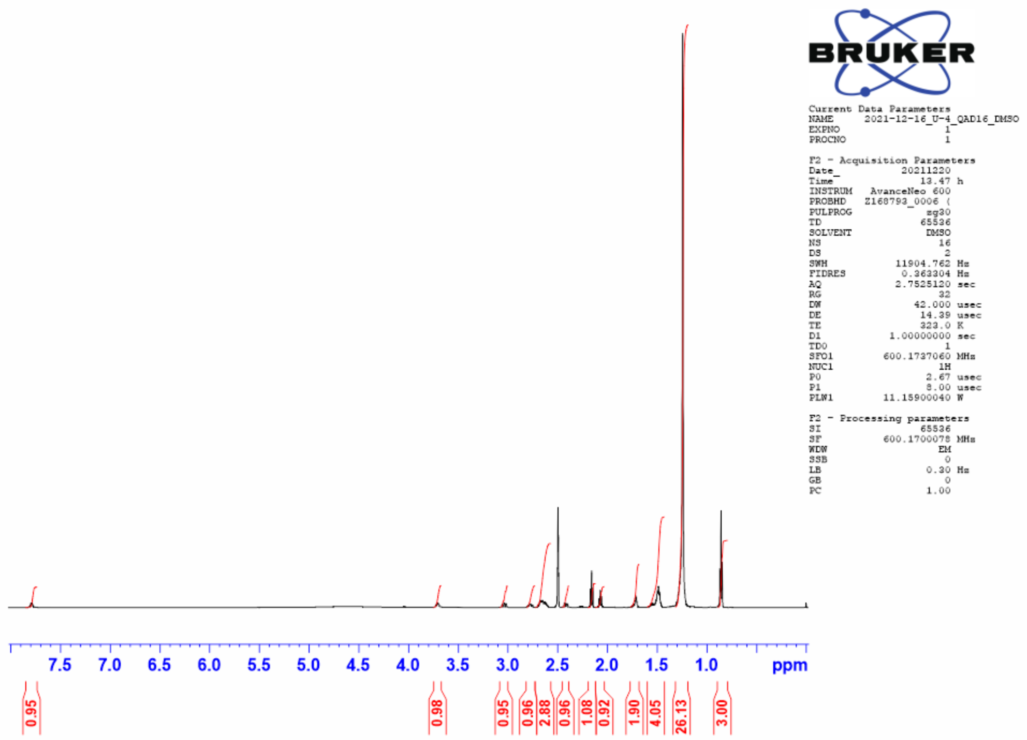

^{13}C NMR spectrum of **QC_{16}**

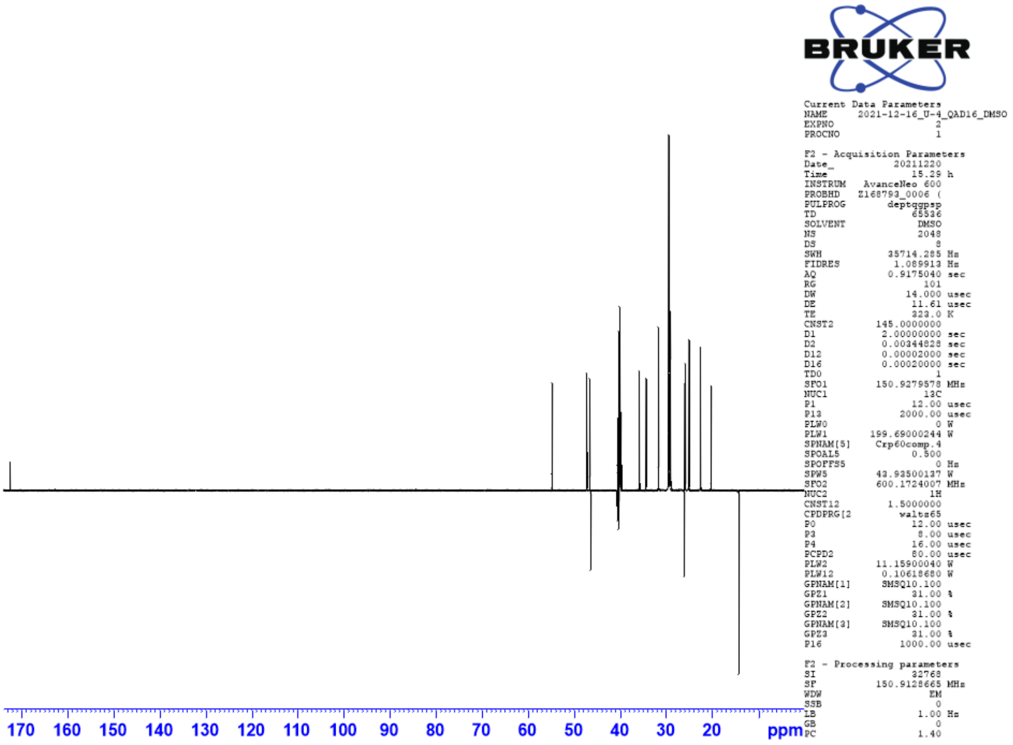

^{1}H NMR spectrum of **QC_{12}-Me**

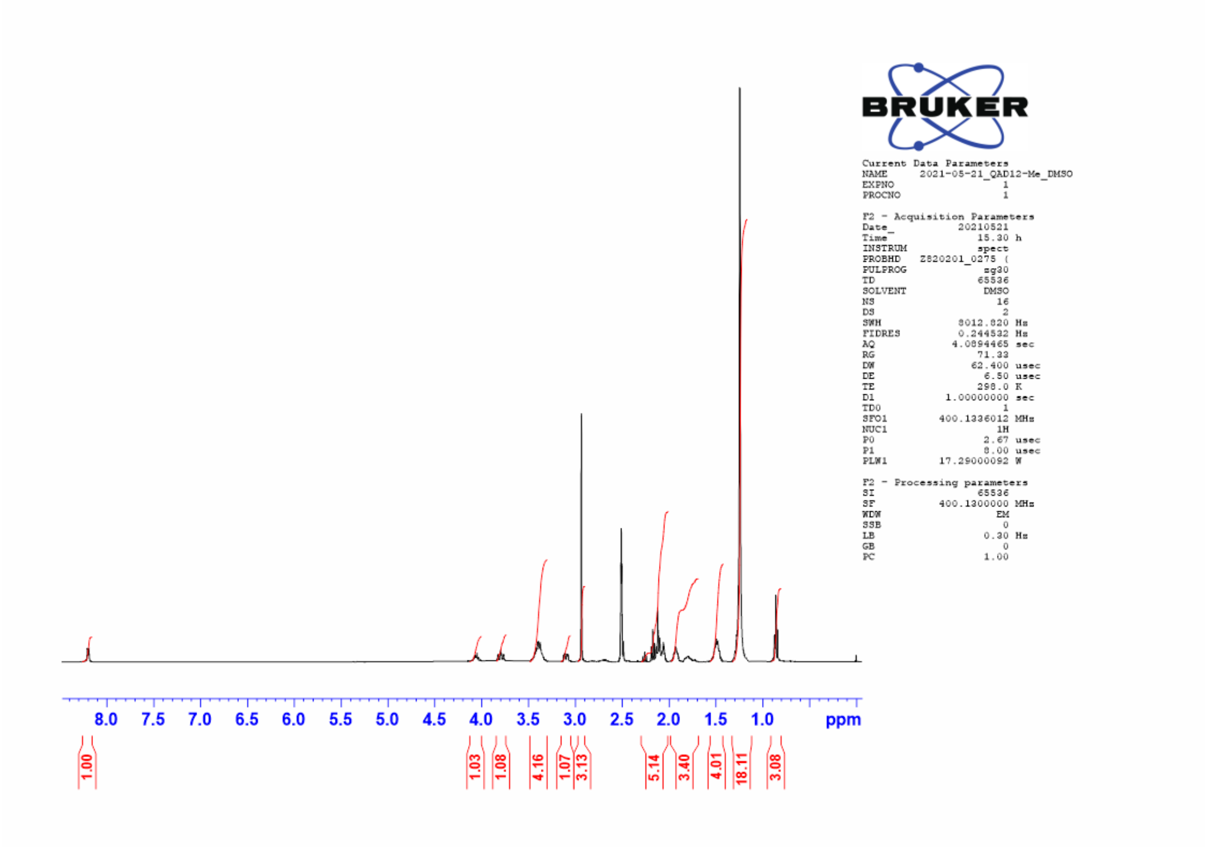

^{13}C NMR spectrum of **QC_{12}-Me**

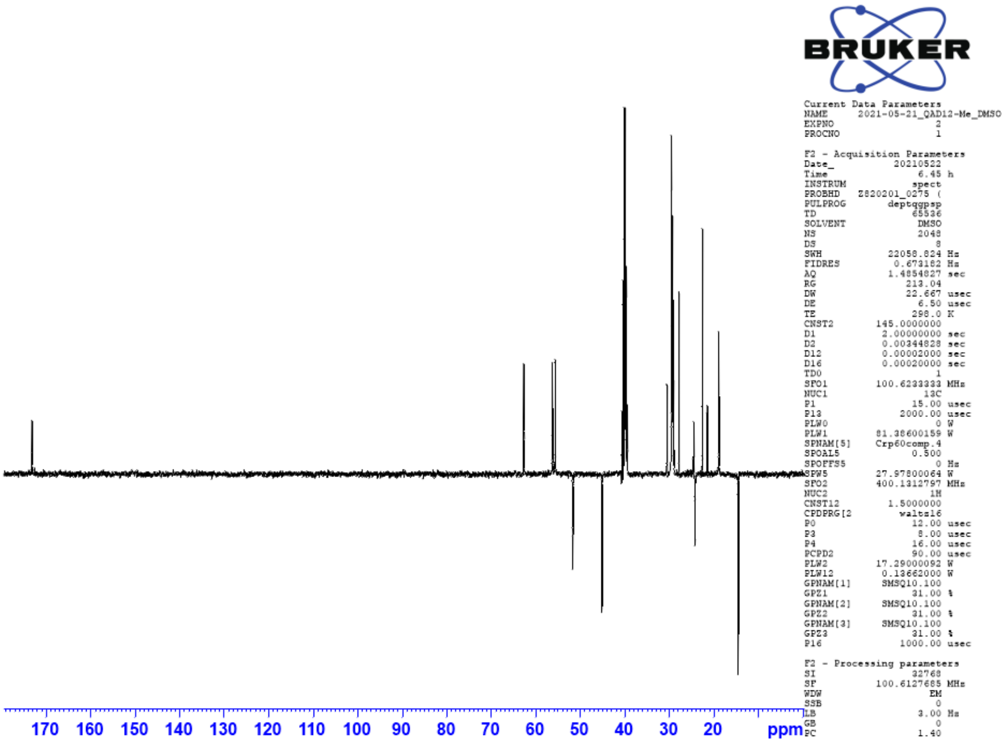

^{1}H NMR spectrum of **QC_{14}-Me**

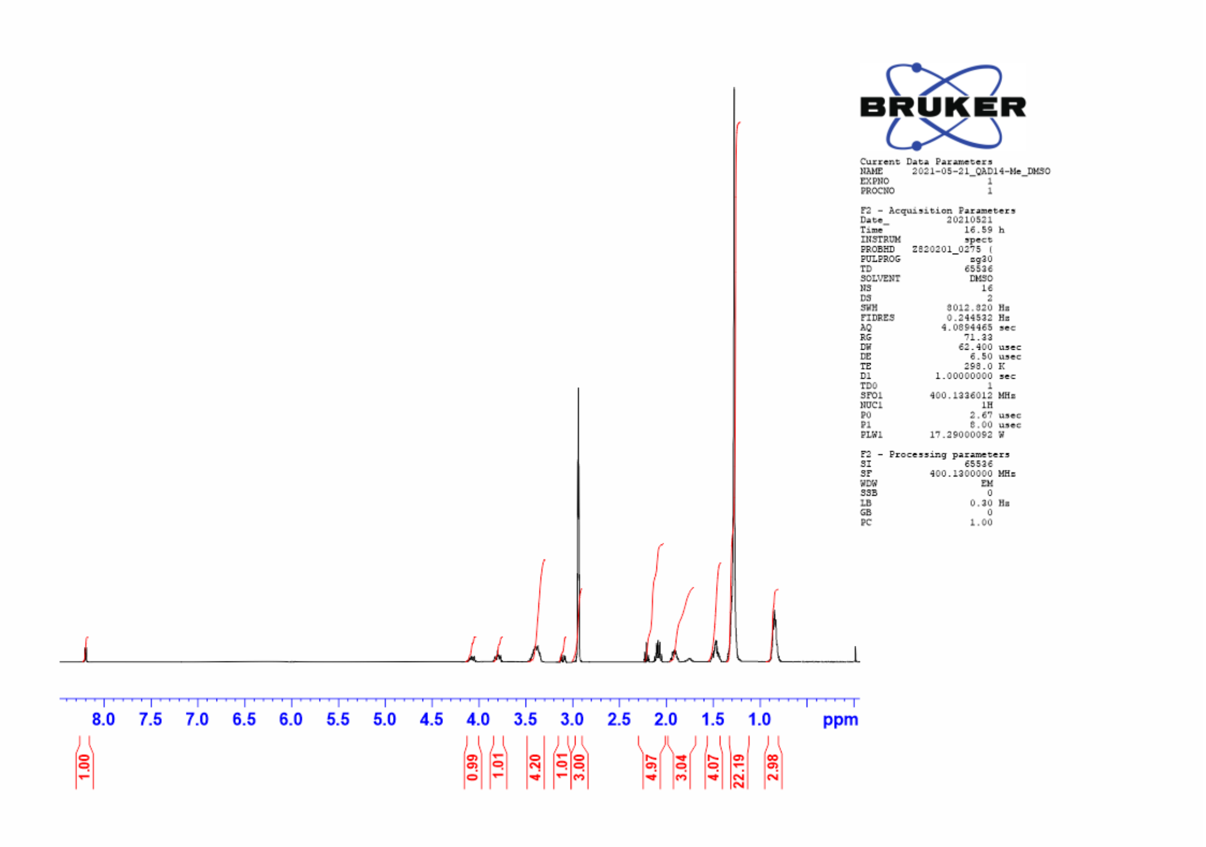

^{13}C NMR spectrum of **QC$_{14}$-Me**

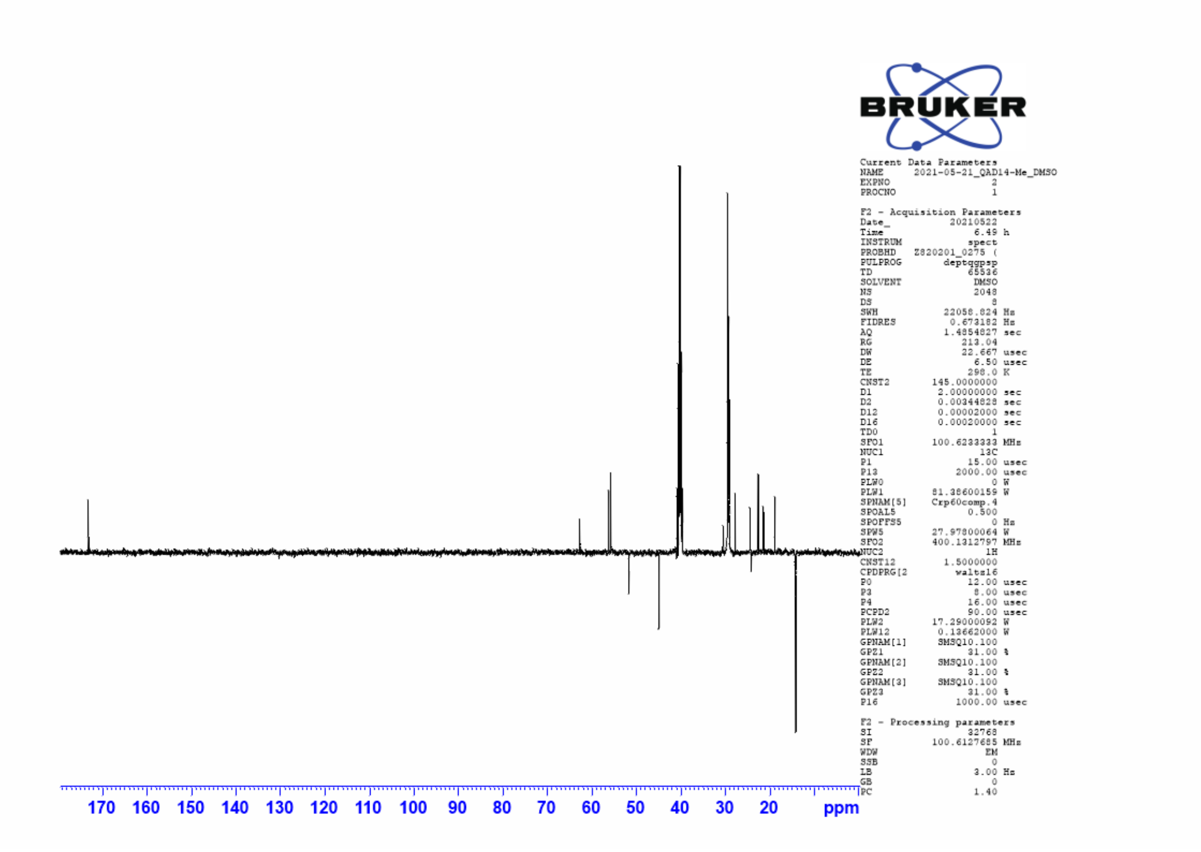

^{1}H NMR spectrum of **QC_{16}-Me**

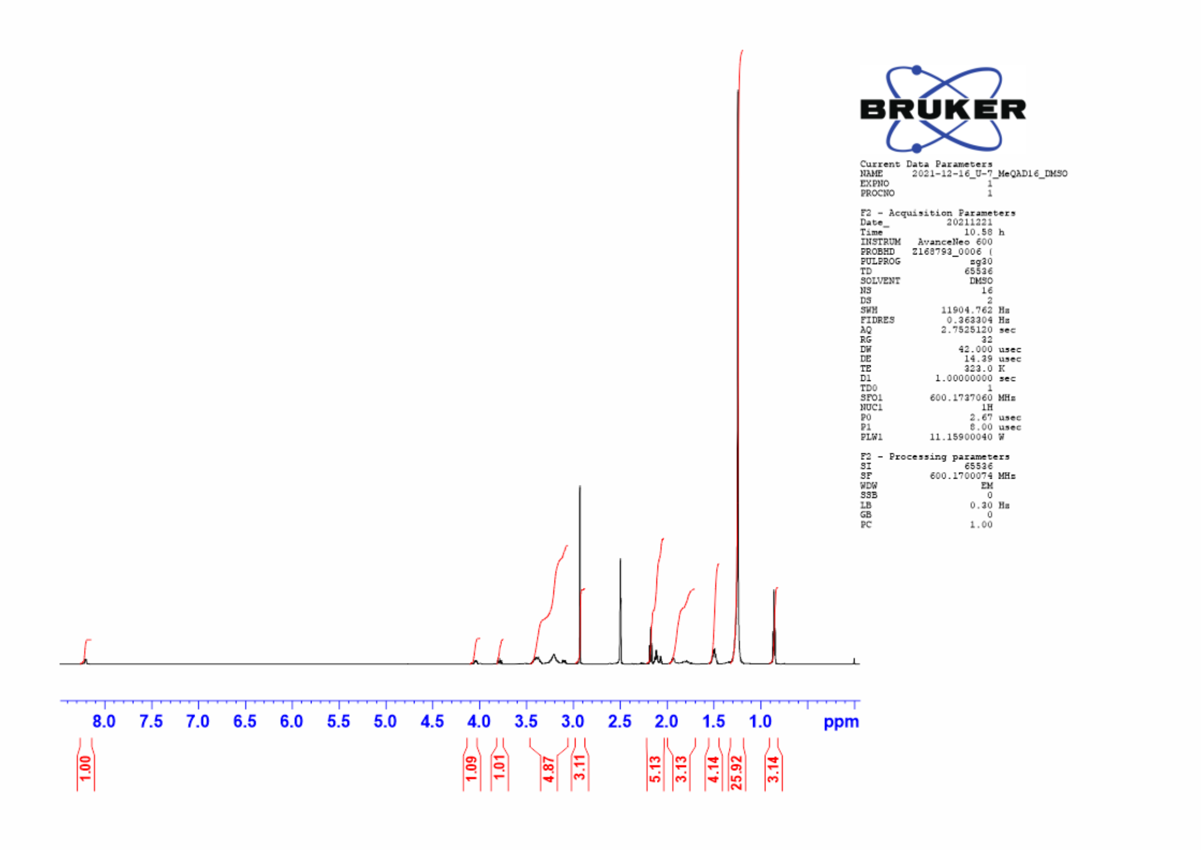

^{13}C NMR spectrum of **QC_{16}-Me**

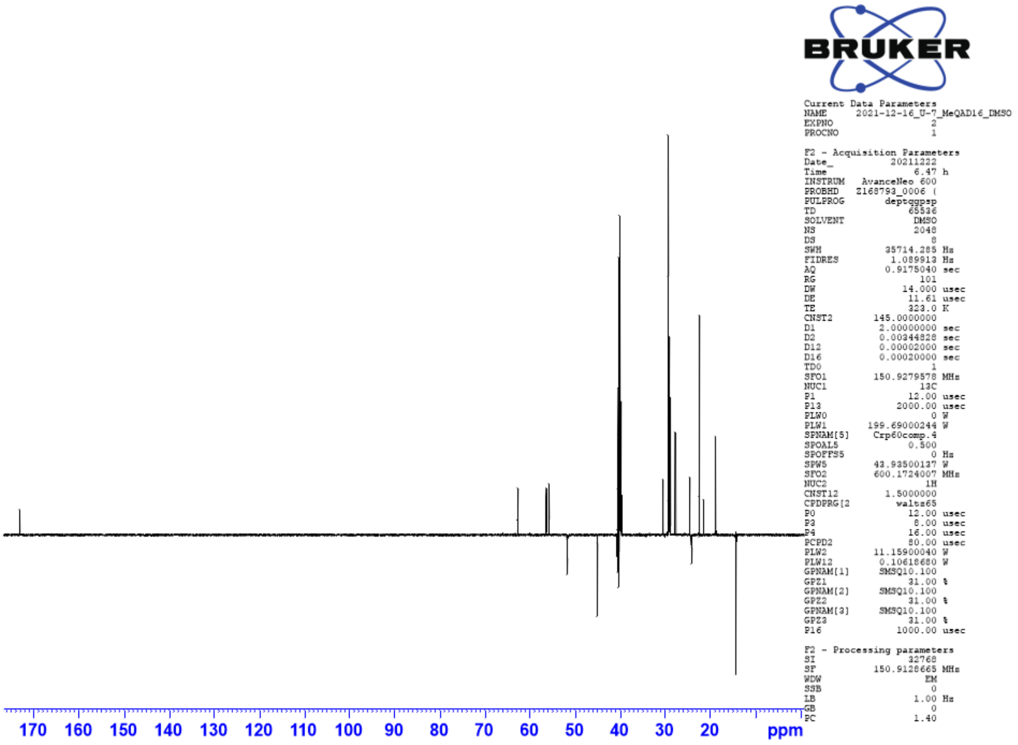

^{1}H NMR spectrum of **QC_{12}-Ally**

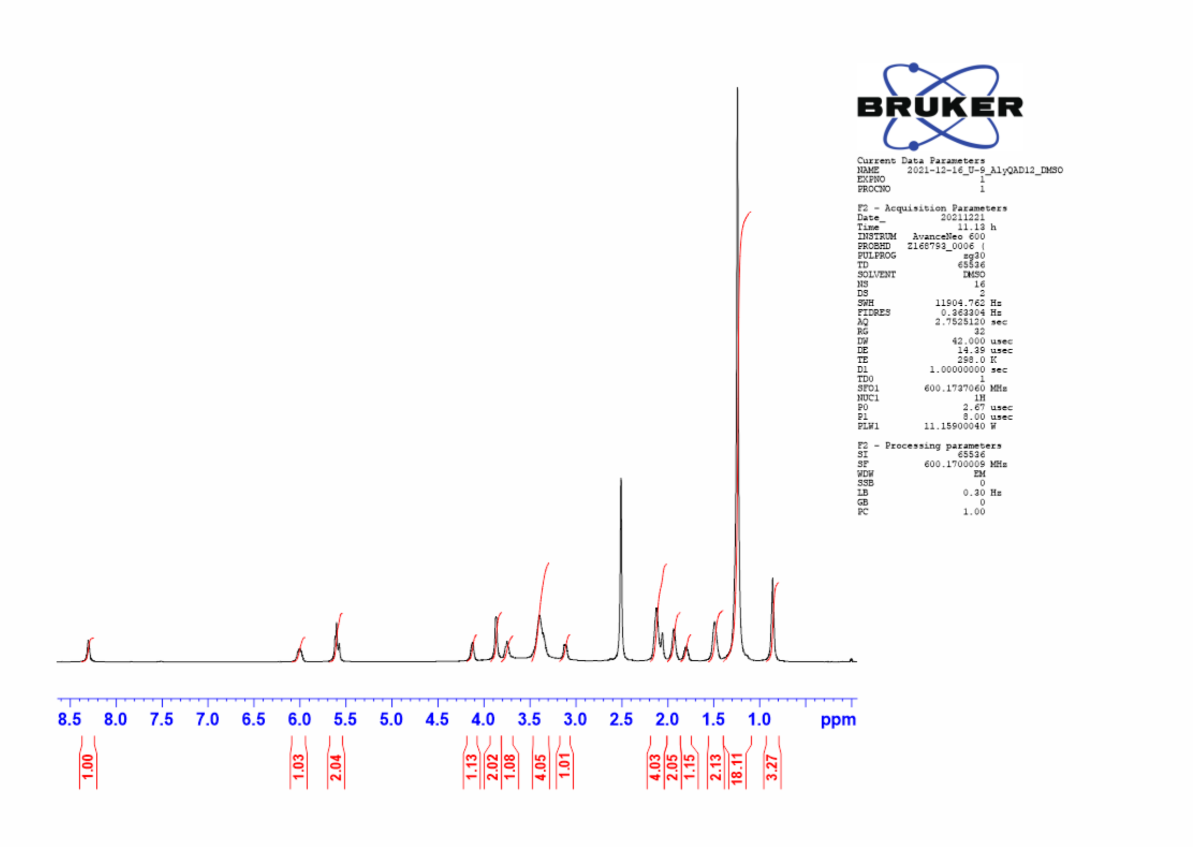

^{13}C NMR spectrum of **QC_{12}-Ally**

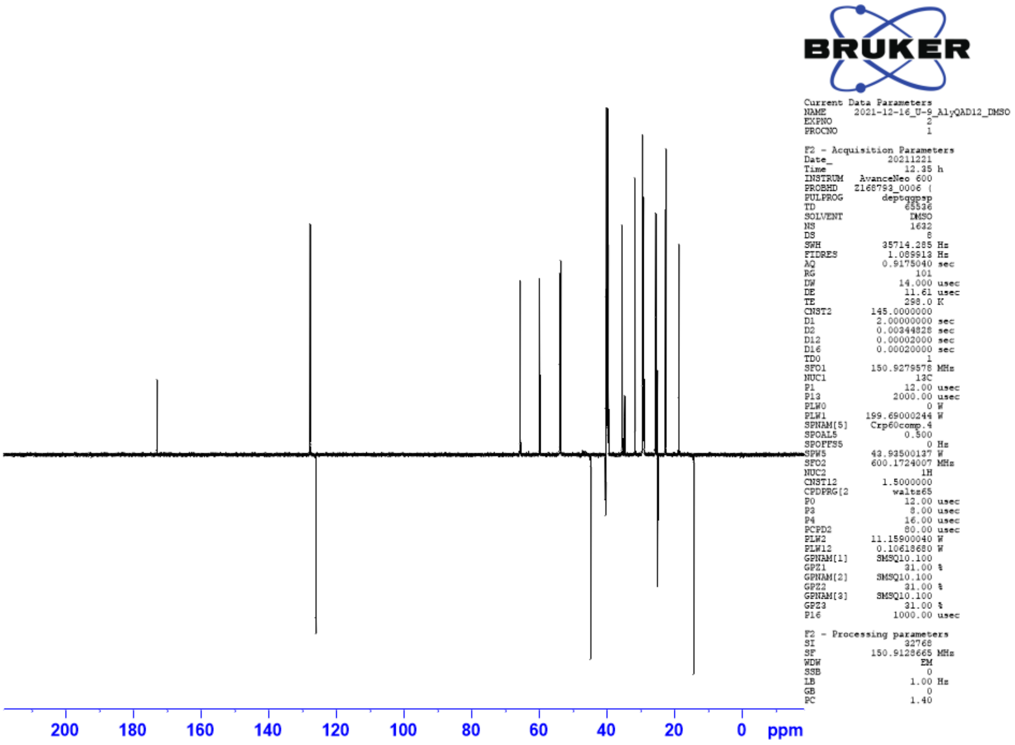

^{1}H NMR spectrum of **QC14-Ally**

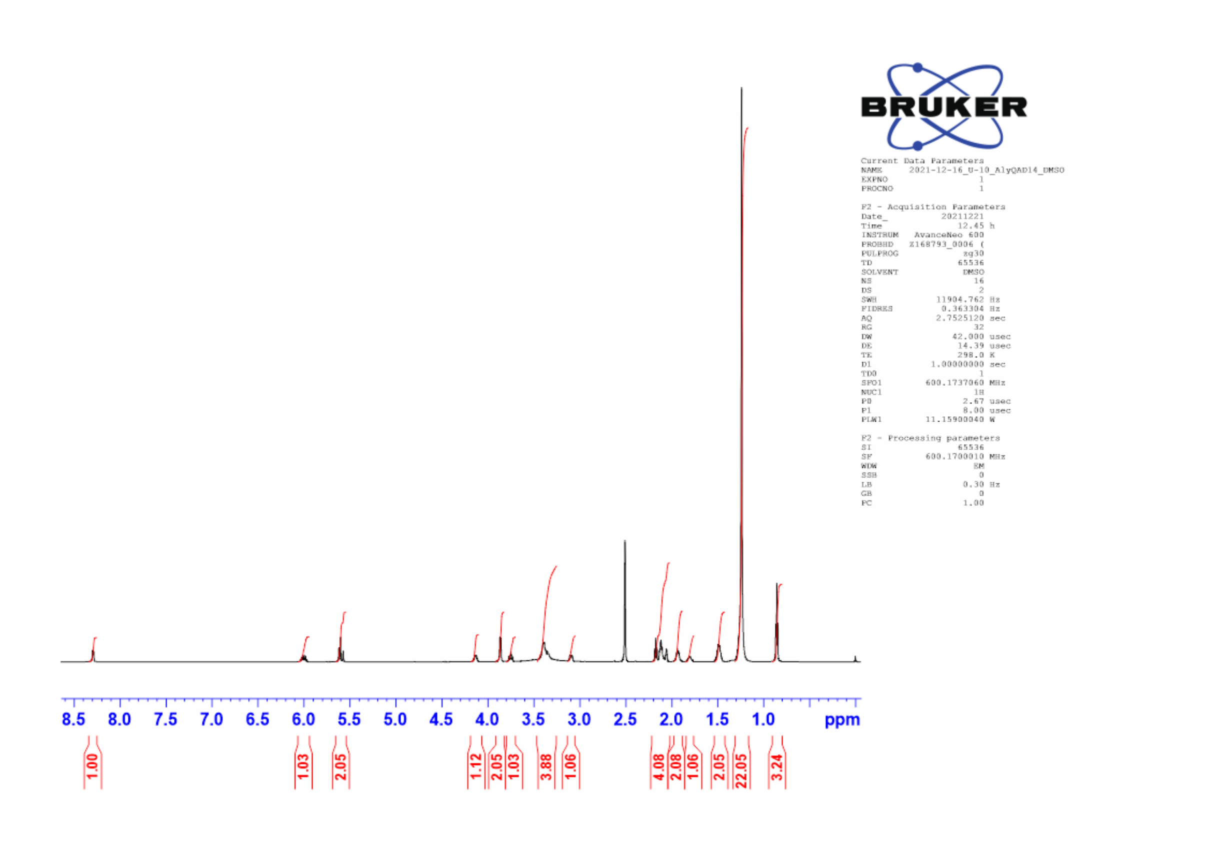

^{13}C NMR spectrum of **QC_{14}-Ally**

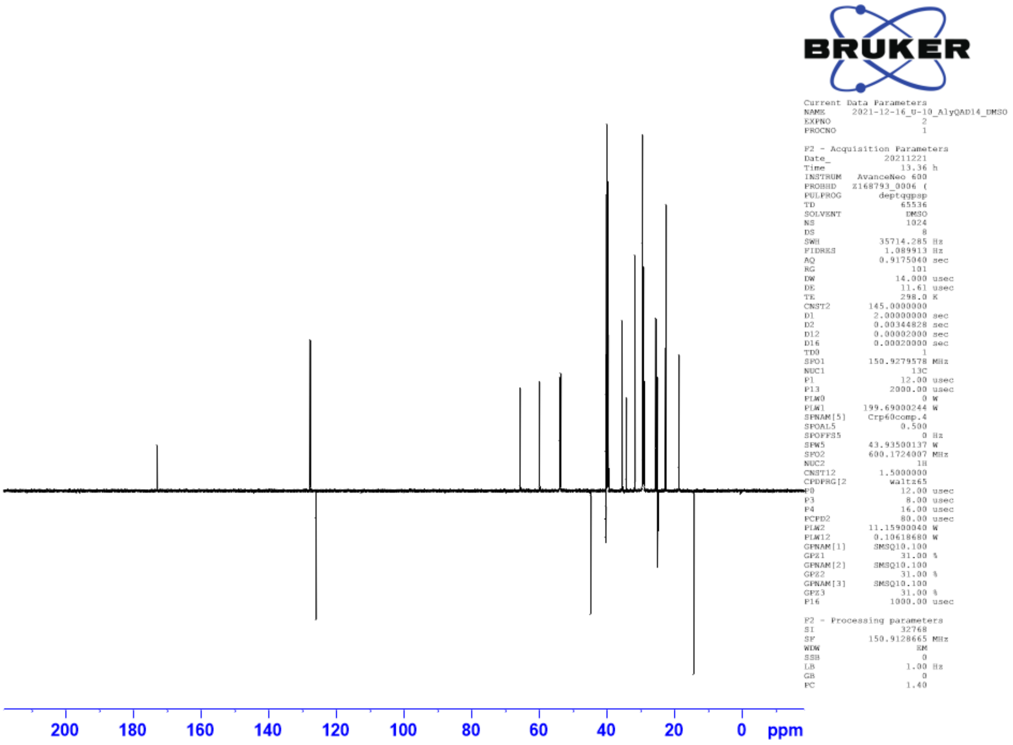

^{1}H NMR spectrum of **QC_{16}-Ally**

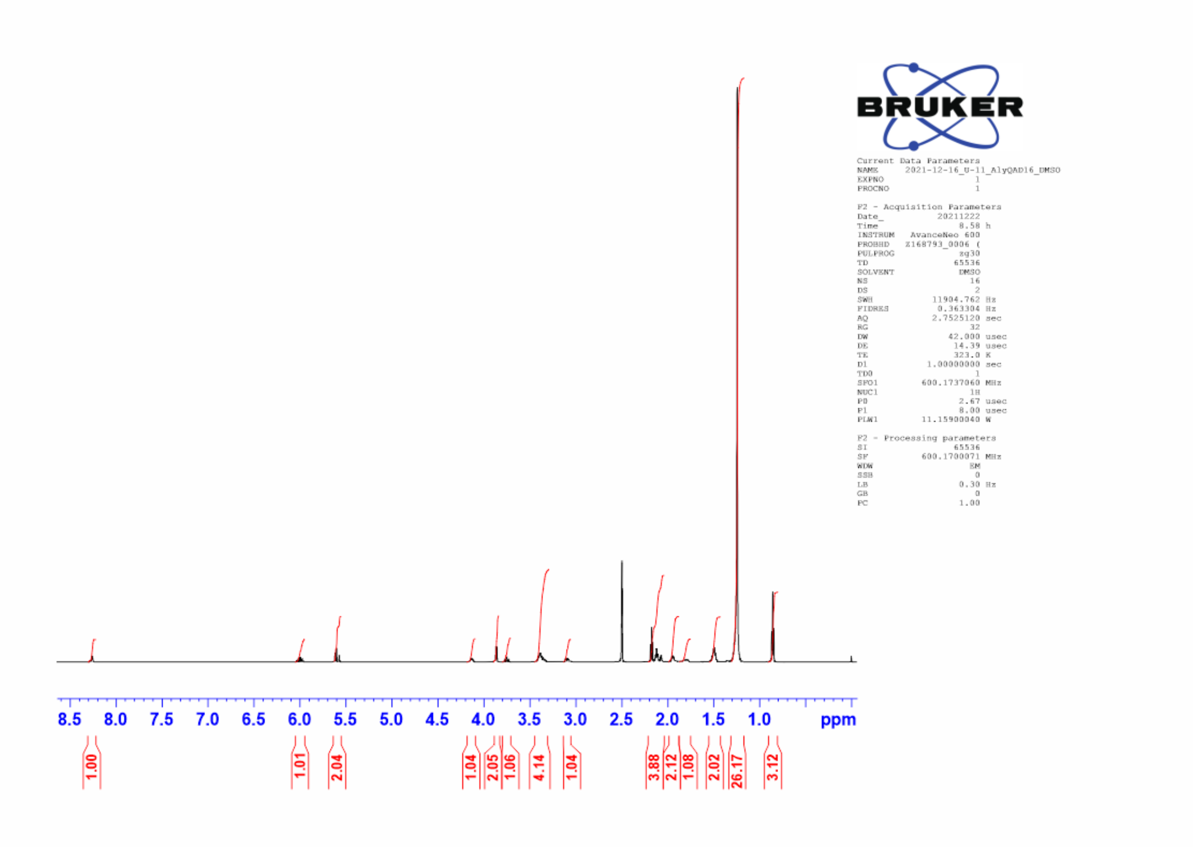

^{13}C NMR spectrum of **QC$_{16}$-Ally**

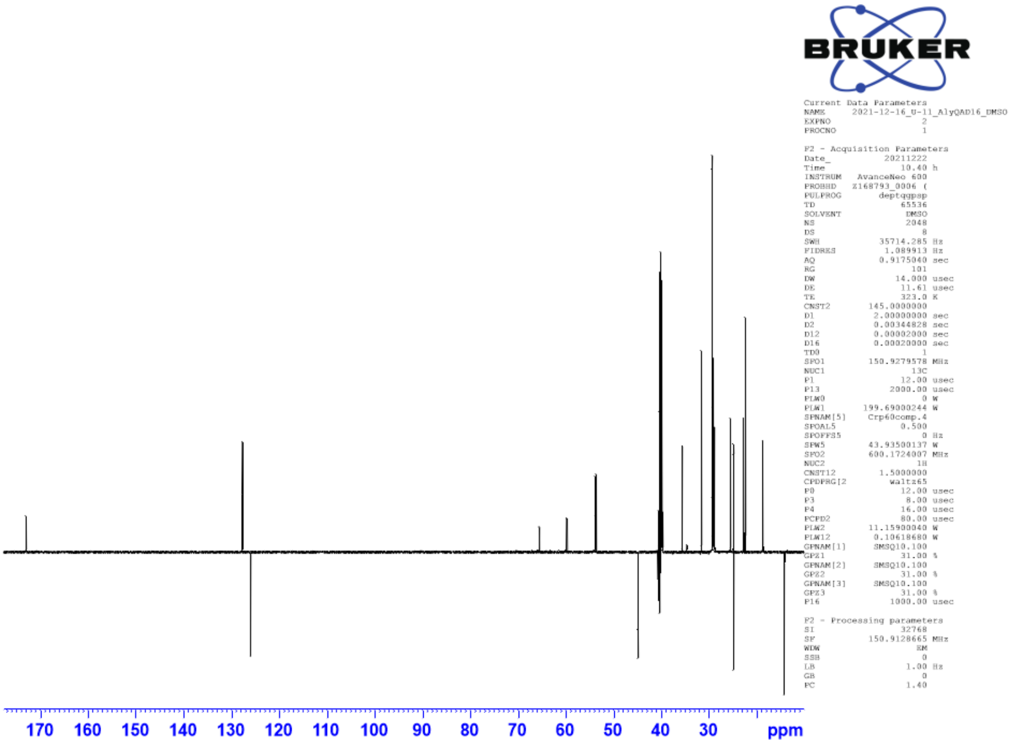

^{1}H NMR spectrum of **QAc-C12**

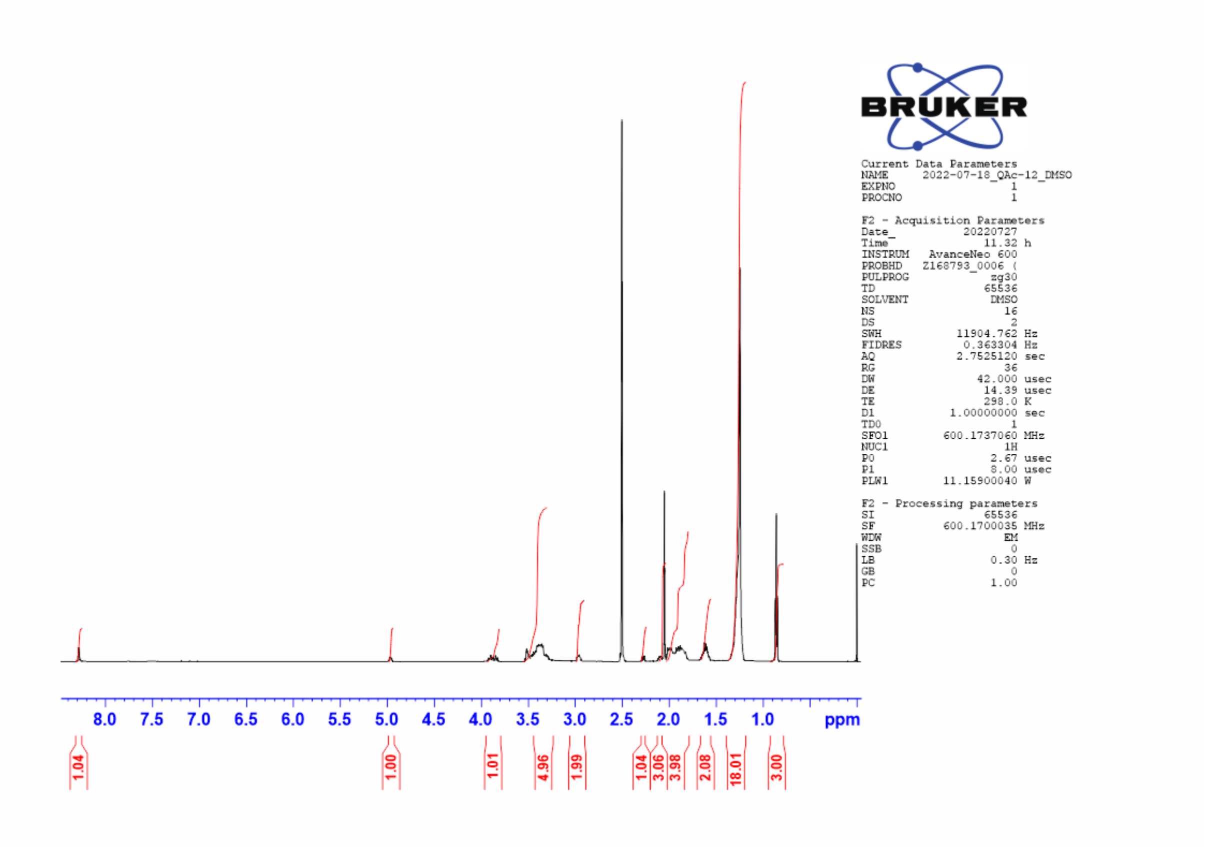

^{13}C NMR spectrum of **QAc-C$_{12}$**

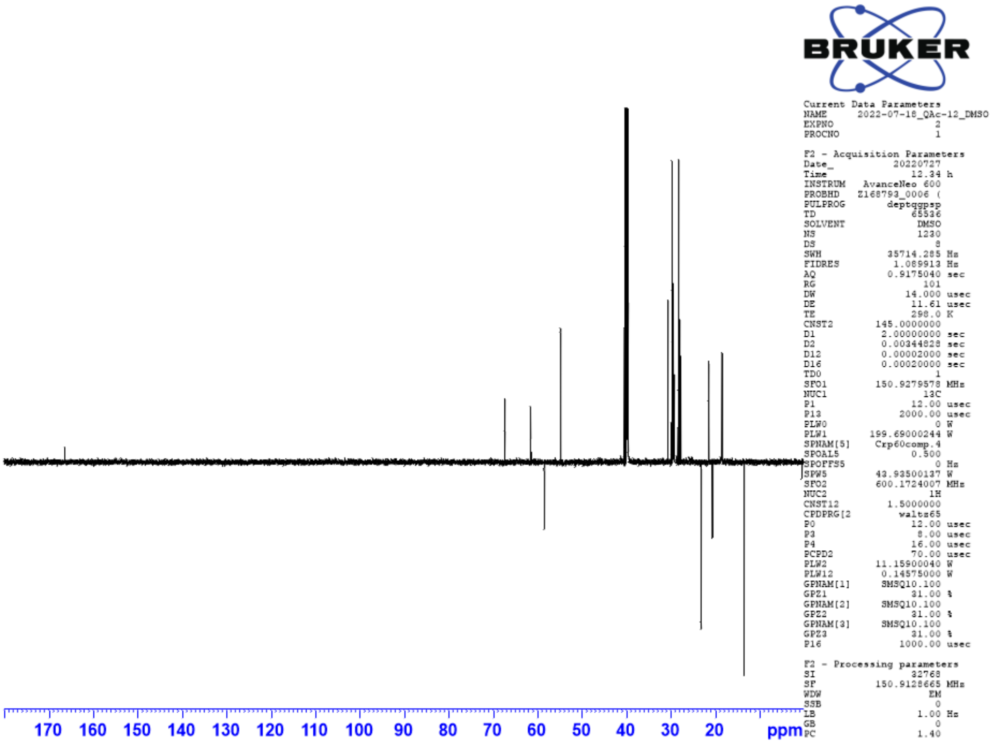

^{1}H NMR spectrum of **QAc-C$_{14}$**

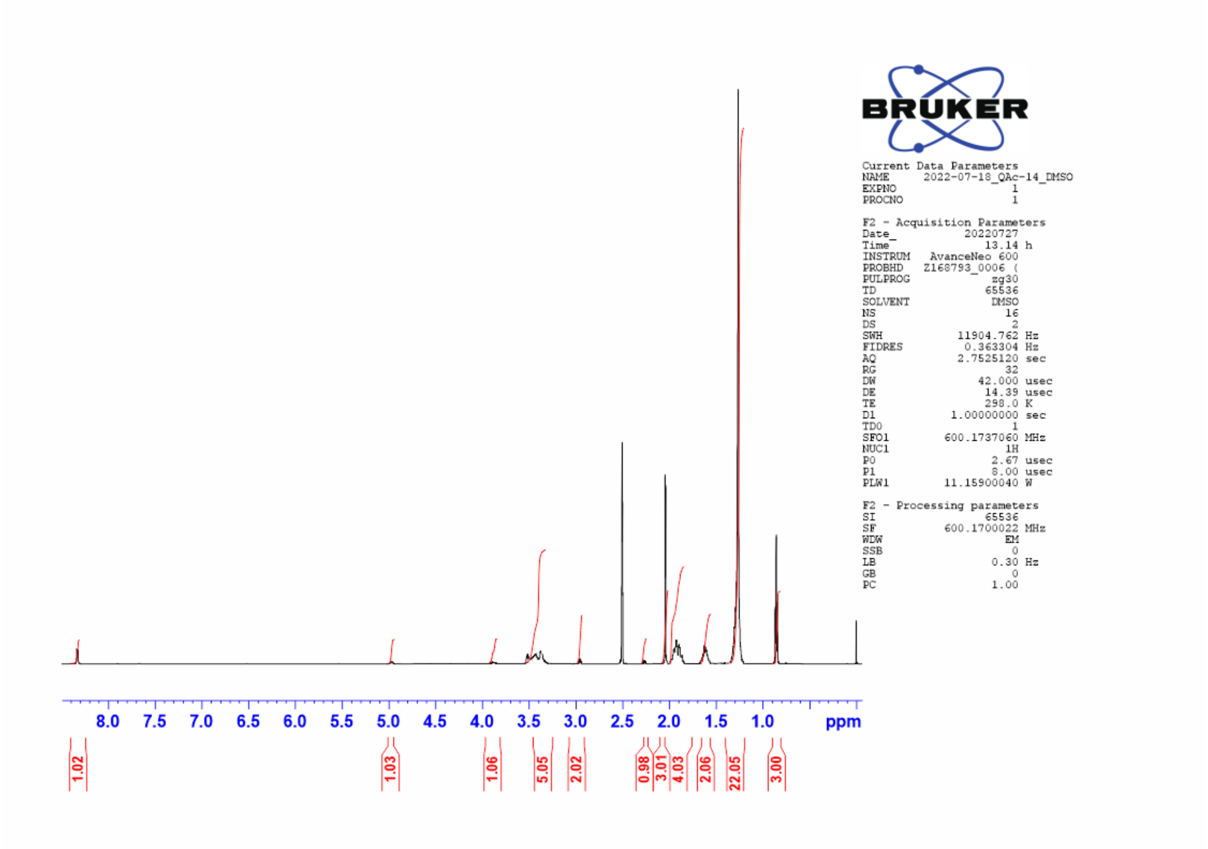

^{13}C NMR spectrum of **QAc-C$_{14}$**

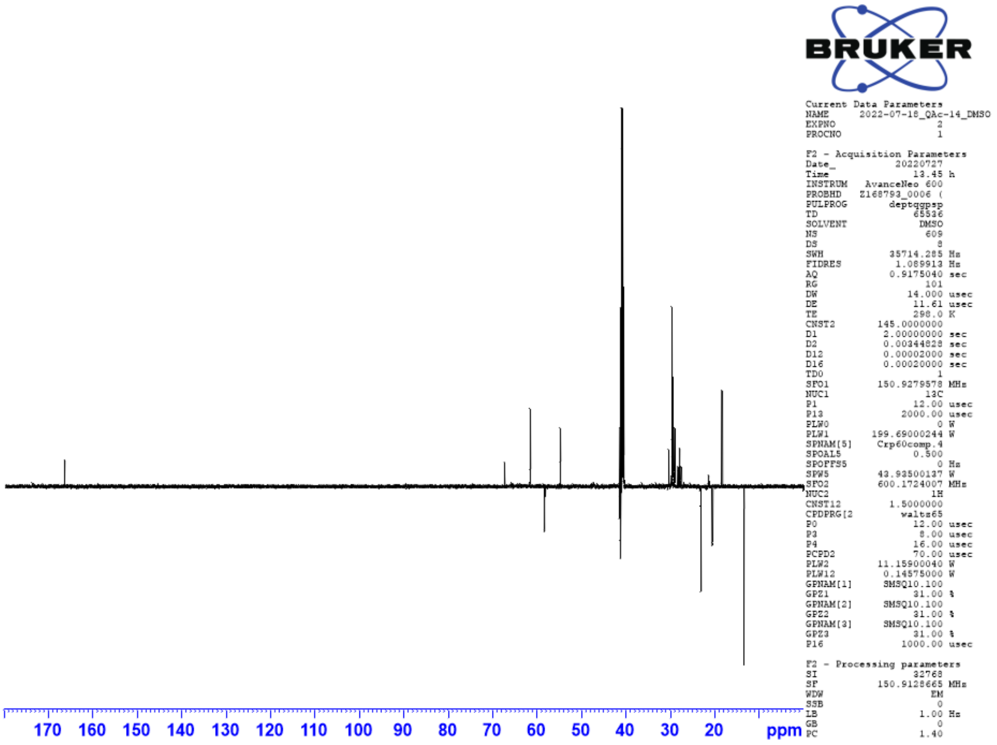

^{1}H NMR spectrum of **QAc-C16**

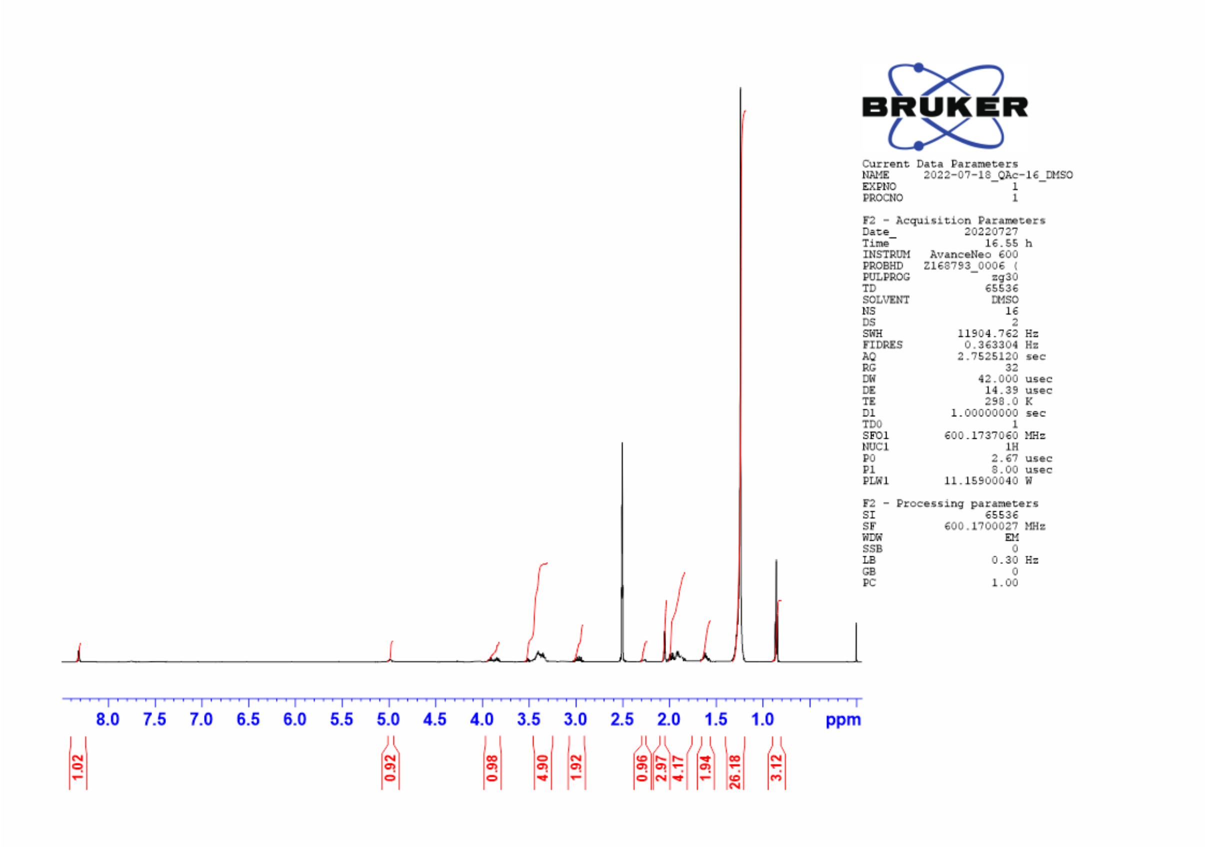

^{13}C NMR spectrum of **QAc-C_{16}**

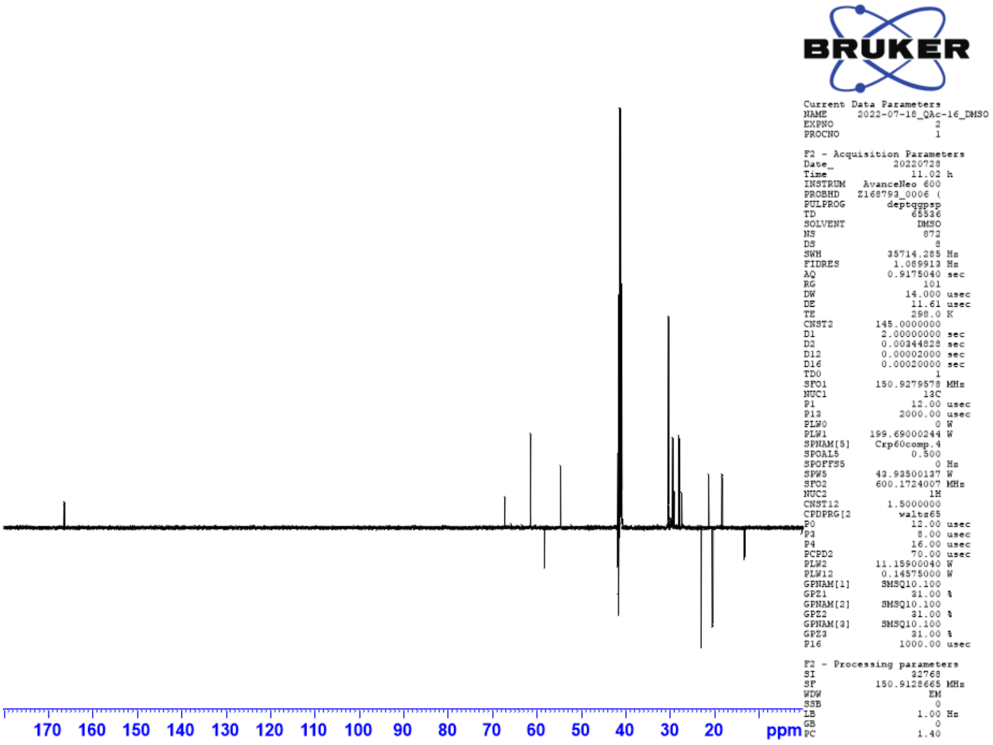

^{1}H NMR spectrum of **QBn-C12**

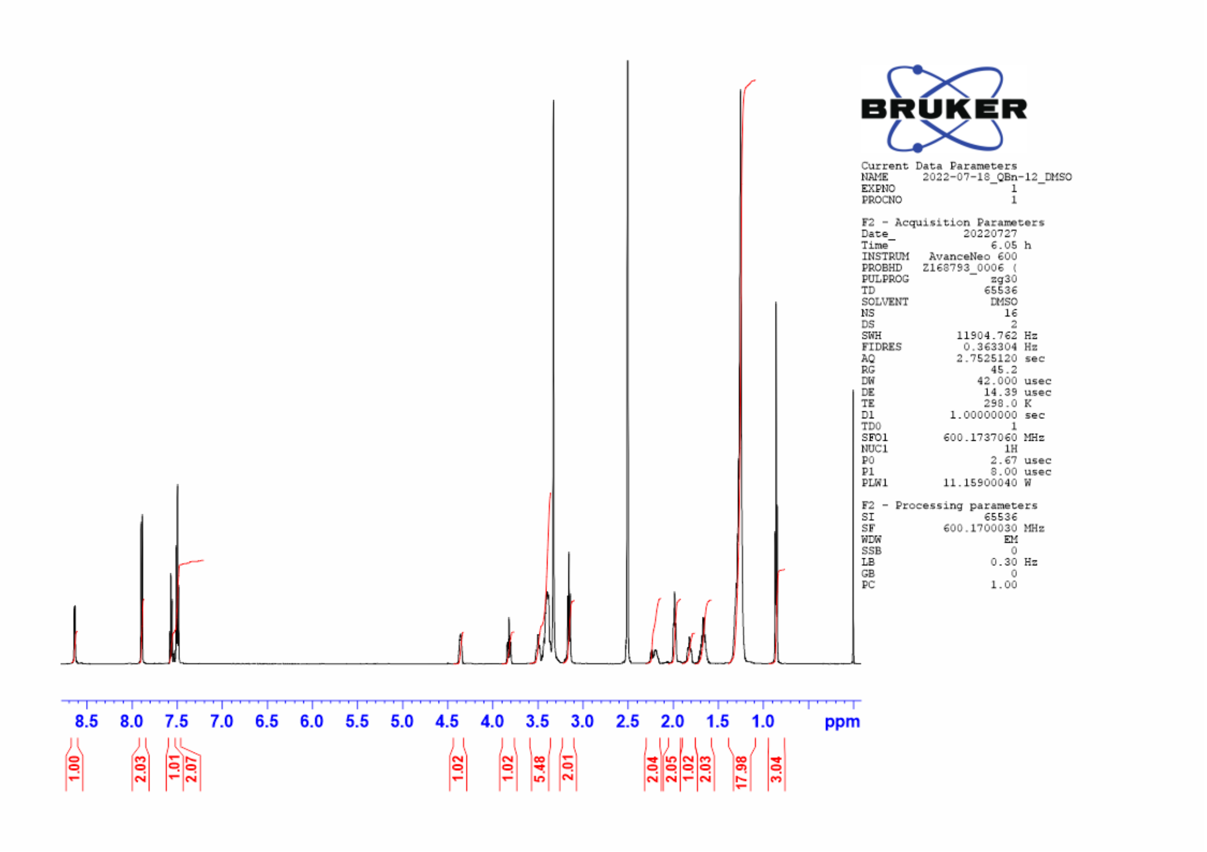

^{13}C NMR spectrum of **QBn-C12**

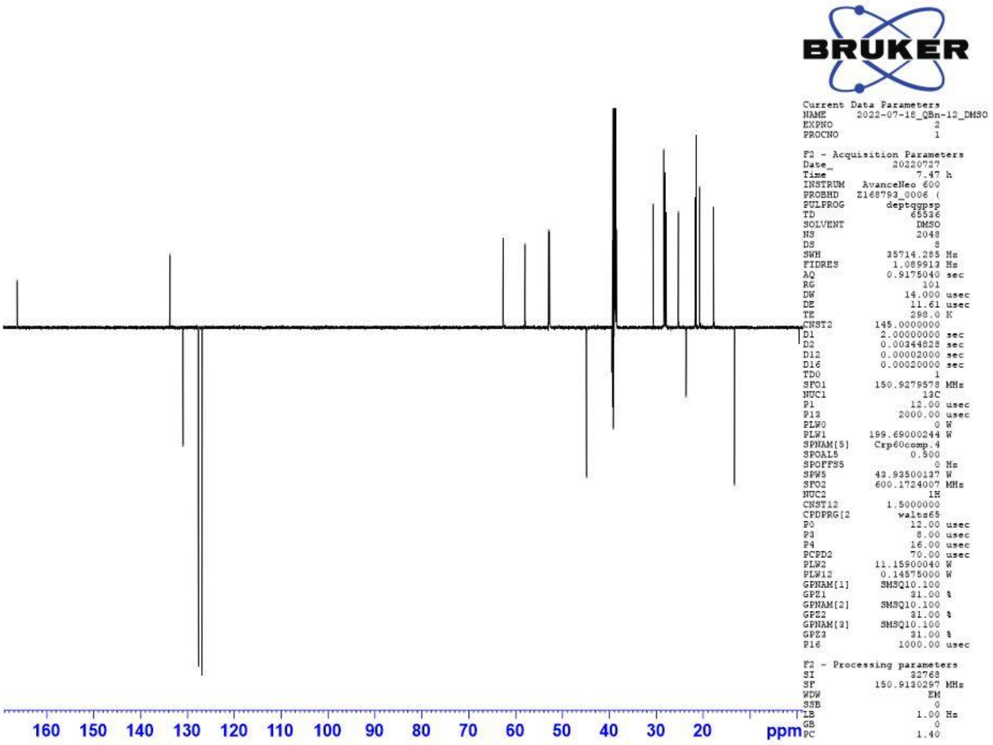

^{1}H NMR spectrum of **QBn-C$_{14}$**

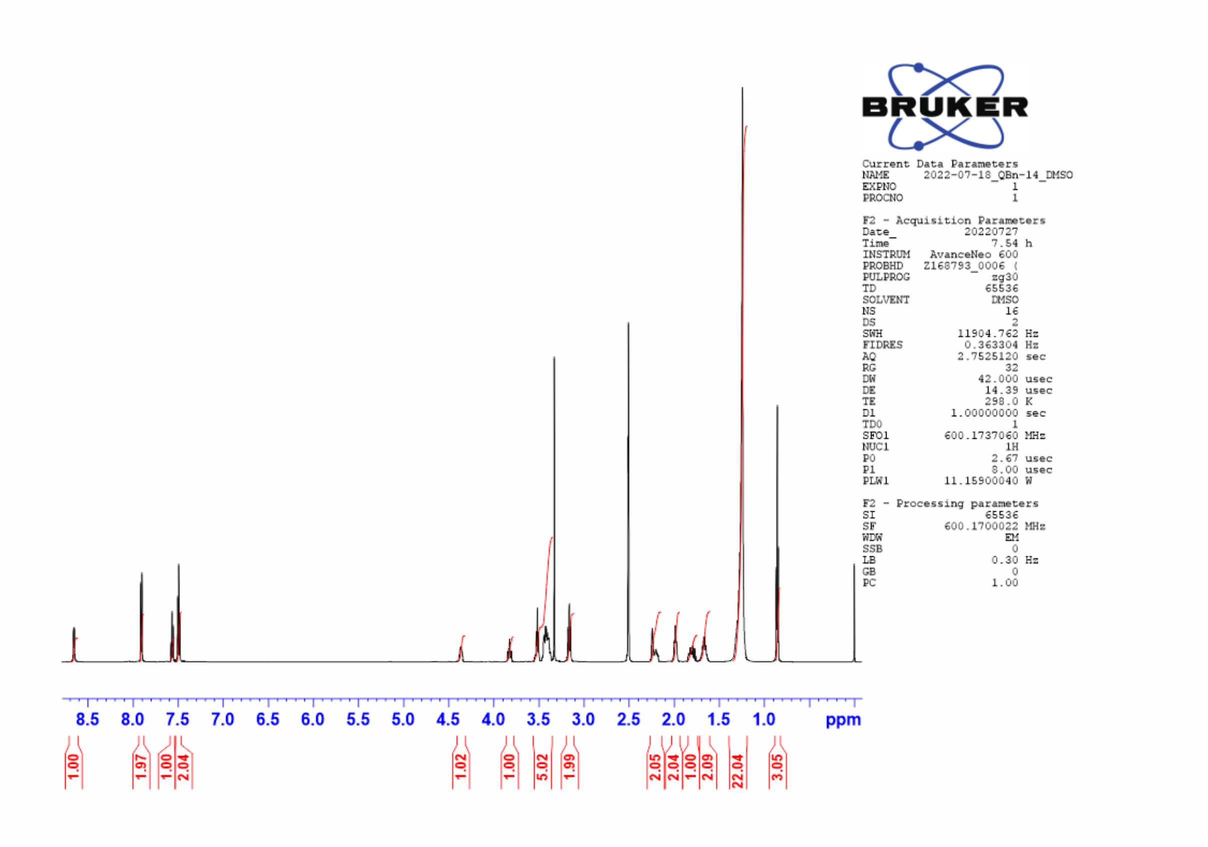

^{13}C NMR spectrum of **QBn-C$_{14}$**

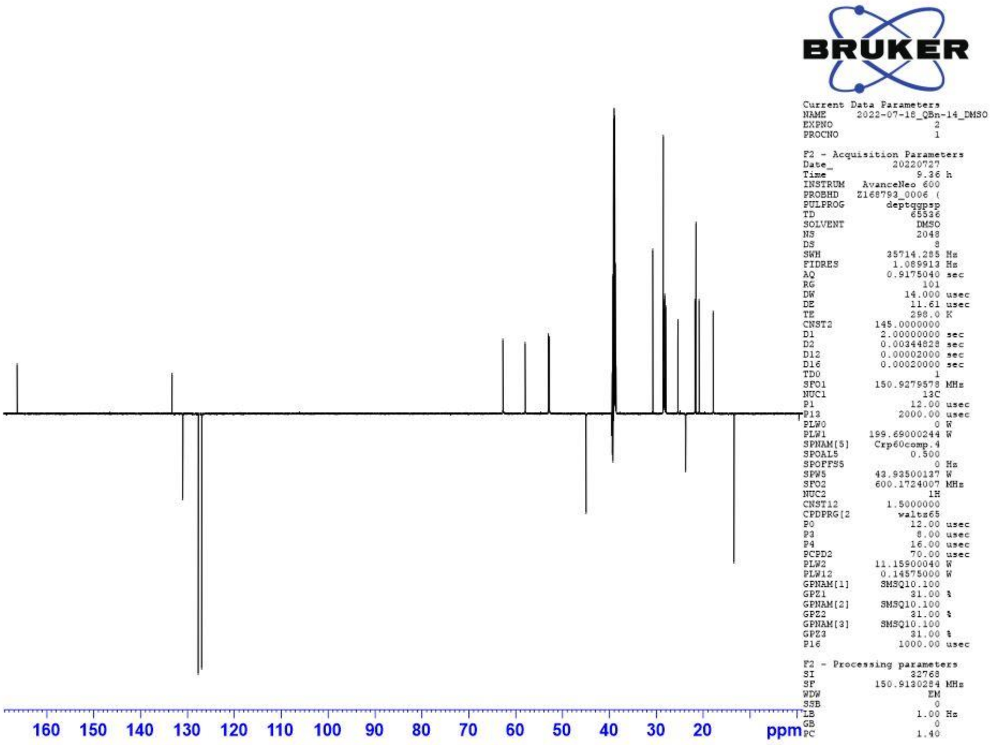

^{1}H NMR spectrum of **QBn-C16**

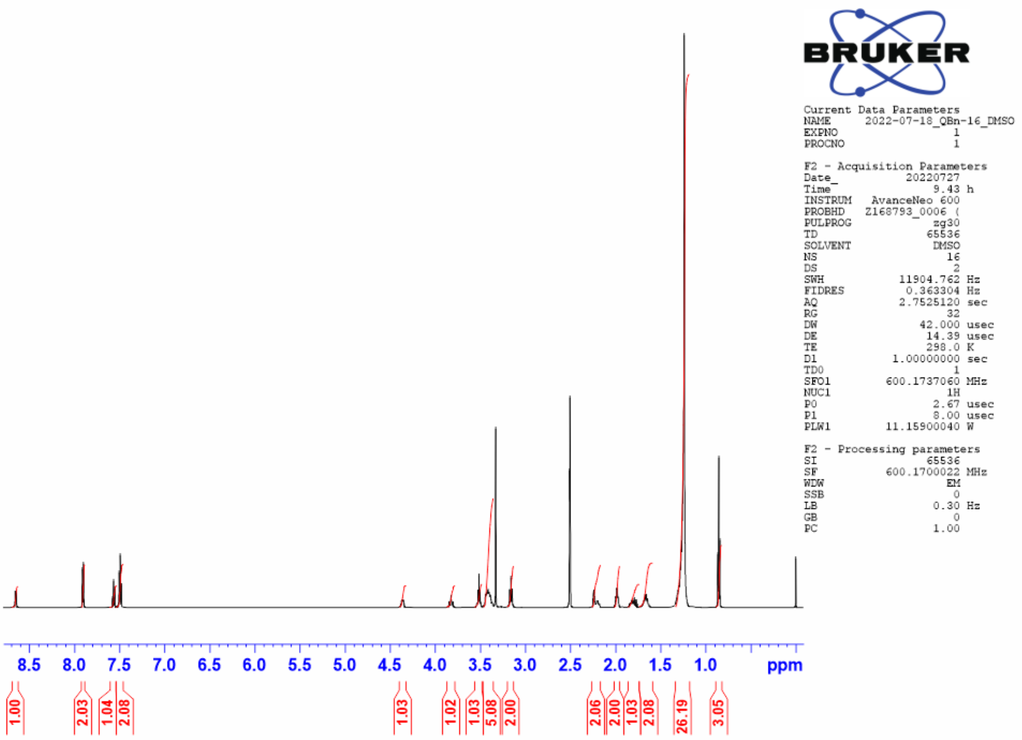

^{13}C NMR spectrum of **QBn-C$_{16}$**

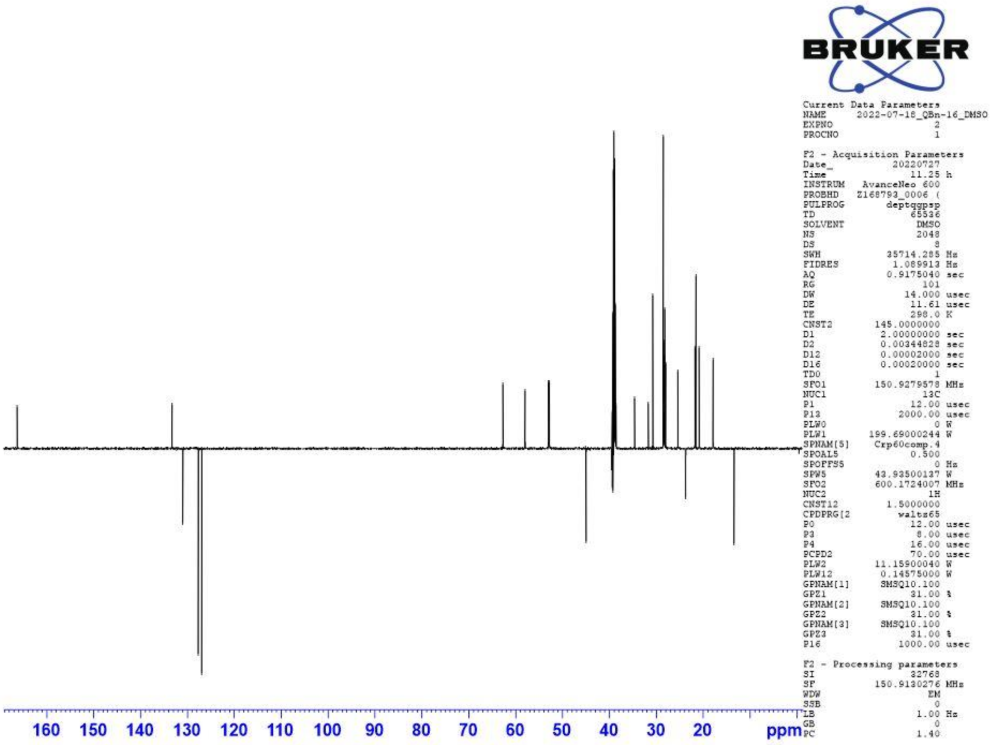

(±) 3-dodecylamidoquinuclidine (**QC$_{12}$**): ^{1}H NMR (DMSO-d$_6$) δ/ppm: 0.86 (t, *J* = 6.96 Hz, 3H, H12'), 1.18–1.32 (m, 18H, H3'–H11', H5 *trans*), 1.43–1.57 (m, 4H, H2', H8), 1.67–1.75 (m, 2H, H4 and H5 *cis*), 2.04–2.17 (m, 2H, H1'), 2.37–2.43 (m, 1H, H2 *trans*), 2.55–2.79 (m, 4H, H6 and H7), 2.99–3.07 (m, 1H, H2 *cis*), 3.66–3.74 (m, 1H, H3), 7.80 (d, *J* = 6.97 Hz, 1H, CONH).
^{13}C NMR (DMSO-d$_6$) δ/ppm: 14.41 (C12'), 20.19, 22.57, 25.14, 25.87, 29.04–29.54, 31.76, 34.56, 35.77 (C1'–C11', C5, C8), 26.10 (C4), 46.36 (C3), 46.62 (C6), 47.22 (C7), 54.74 (C2), 172.50 (C=O).

(±) 3-tetradecylamidoquinuclidine (**QC$_{14}$**): ^{1}H NMR (DMSO-d$_6$) δ/ppm: 0.85 (t, *J* = 6.97 Hz, 3H, H14'), 1.20–1.33 (m, 22H, H3'–H11', H5 *trans*), 1.44–1.57 (m, 4H, H2', H8), 1.66–1.76 (m, 2H, H4 and H5 *cis*), 2.03–2.19 (m, 2H, H1'), 2.36–2.45 (m, 1H, H2 *trans*), 2.54–2.79 (m, 4H, H6 and H7), 3.00–3.05 (m, 1H, H2 *cis*), 3.65–3.72 (m, 1H, H3), 7.81 (d, *J* = 6.98 Hz, 1H, CONH).
^{13}C NMR (DMSO-d$_6$) δ/ppm: 14.40 (C14'), 20.20, 22.56, 25.16, 25.86, 29.05–29.58, 31.74, 34.55, 35.76 (C1'–C13', C5, C8), 26.11 (C4), 46.38 (C3), 46.64 (C6), 47.23 (C7), 54.73 (C2), 172.51 (C=O).

(±) 3-hexadecylamidoquinuclidine (**QC$_{16}$**): ^{1}H NMR (DMSO-d$_6$) δ/ppm: 0.85 (t, *J* = 6.96 Hz, 3H, H16'), 1.20–1.32 (m, 26H, H3'–H11', H5 *trans*), 1.42–1.57 (m, 4H, H2', H8), 1.68–1.75 (m, 2H, H4 and H5 *cis*), 2.03–2.18 (m, 2H, H1'), 2.37–2.44 (m, 1H, H2 *trans*), 2.56–2.77 (m, 4H, H6 and H7), 2.97–3.08 (m, 1H, H2 *cis*), 3.64–3.73 (m, 1H, H3), 7.79 (d, *J* = 6.96 Hz, 1H, CONH).
^{13}C NMR (DMSO-d$_6$) δ/ppm: 14.40 (C16'), 20.21, 22.58, 25.15, 25.88, 29.03–29.48, 31.75, 34.57, 35.78 (C1'–C15', C5, C8), 26.10 (C4), 46.37 (C3), 46.63 (C6), 47.22 (C7), 54.75 (C2), 172.52 (C=O).

N-methyl-3-dodecyamidoquinuclidinium iodide (**QC$_{12}$-Me**) ^{1}H NMR (DMSO-d$_6$) δ/ppm: 0.86 (t, *J* = 6.61 Hz, 3H, H12'), 1.20–1.37 (m, 18H, H3'–H11'), 1.43–1.54 (m, 4H, H2', H8), 1.71–1.96 (m, 3H, H4 and H5), 2.04–2.28 (m, 5H, H1'), 2.93 (s, 3H, $N^{+}CH_3$), 3.07–3.13 (m, 1H, H2 *trans*), 3.31–3.47 (m, 4H, H6, H7), 3.76–3.83 (m, 1H, H2 *cis*), 4.03–4.11 (m, 1H, H3), 8.20 (d, *J* = 5.67 Hz, 1H, CONH).

^{13}C NMR (DMSO-d_6) δ/ppm: 14.41 (C12'), 18.82, 21.49, 21.68, 22.25, 24.59, 27.91, 28.99–29.62, 30.69 (C1'–C11', C5, C8), 24.13 (C4), 45.06 (N^+CH3), 51.65 (C3), 55.62 (C6), 56.19 (C7), 62.62 (C2), 173.17 (C=O).

N-methyl-3-tetradecyamidoquinuclidinium iodide (**QC$_{14}$-Me**) ^{1}H NMR (DMSO-d_6) δ/ppm: 0.86 (t, *J* = 6.60 Hz, 3H, H14'), 1.22–1.36 (m, 22H, H3'–H11'), 1.41–1.54 (m, 4H, H2', H8), 1.71–1.97 (m, 3H, H4 and H5), 2.03–2.25 (m, 5H, H1'), 2.94 (s, 3H, N^+CH_3), 3.08–3.13 (m, 1H, H2 *trans*), 3.31–3.49 (m, 4H, H6, H7), 3.74–3.84 (m, 1H, H2 *cis*), 4.05–4.12 (m, 1H, H3), 8.21 (d, *J* = 5.66 Hz, 1H, CONH).
^{13}C NMR (DMSO-d_6) δ/ppm: 14.42 (C14'), 18.82, 21.47, 21.70, 22.24, 24.58, 27.90, 29.00–29.61, 30.70 (C1'–C13', C5, C8), 24.14 (C4), 45.04 (N^+CH3), 51.66 (C3), 55.64 (C6), 56.20 (C7), 62.63 (C2), 173.18 (C=O).

N-methyl-3-hexadecyamidoquinuclidinium iodide (**QC$_{16}$-Me**) ^{1}H NMR (DMSO-d_6) δ/ppm: 0.87 (t, *J* = 6.61 Hz, 3H, H16'), 1.21–1.37 (m, 26H, H3'–H11'), 1.44–1.54 (m, 4H, H2', H8), 1.70–1.97 (m, 3H, H4 and H5), 2.02–2.26 (m, 5H, H1'), 2.93 (s, 3H, N^+CH_3), 3.06–3.12 (m, 1H, H2 *trans*), 3.30–3.47 (m, 4H, H6, H7), 3.73–3.81 (m, 1H, H2 *cis*), 4.04–4.09 (m, 1H, H3), 8.21 (d, *J* = 5.67 Hz, 1H, CONH).
^{13}C NMR (DMSO-d_6) δ/ppm: 14.42 (C16'), 18.84, 21.48, 21.69, 22.25, 24.58, 27.92, 28.97–29.63, 30.67 (C1'–C15', C5, C8), 24.15 (C4), 45.07 (N^+CH3), 51.65 (C3), 55.64 (C6), 56.21 (C7), 62.63 (C2), 173.16 (C=O).

N-allyl-3-dodecylamidoquinuclidinium bromide (**QC$_{12}$-Ally**) ^{1}H NMR (DMSO-d_6) δ/ppm: 0.86 (t, *J* = 6.85 Hz, 3H, H12'), 1.20–1.30 (m, 18H, H3'–H11'), 1.44–1.53 (m, 2H, H2'), 1.76–1.96 (m, 3H, H4 and H8), 2.04–2.19 (m, 4H, H1', H5), 3.07–3.13 (m, 1H, H2 *trans*), 3.30–3.45 (m, 4H, H6 and H7), 3.72–3.78 (m, 1H, H2 *cis*), 3.85–3.89 (m, 2H, N^+CH_2 ally), 4.10–4.15 (m, 1H, H3), 5.56–5.64 (m, 2H, H3 ally), 5.97–6.07 (m, 1H, H2 ally), 8.28 (d, *J* = 5.97 Hz, 1H, CONH).
^{13}C NMR (DMSO-d_6) δ/ppm: 14.42 (C12'), 18.72, 22.57, 22.69, 25.34, 25.65, 29.09–29.53, 31.76, 34.78, 35.54 (C1'–C11', C5, C8), 24.95 (C4), 44.89 (C3), 53.76 (C6), 53.97 (C7), 59.89 (C2), 65.62 (N^+CH_2 ally), 126.10 (C2 ally), 127.78 (C3 ally), 173.09 (C=O).

N-allyl-3-tetradecylamidoquinuclidinium bromide (**QC$_{14}$-Ally**) ^{1}H NMR (DMSO-d_6) δ/ppm: 0.87 (t, *J* = 6.84 Hz, 3H, H14'), 1.22–1.32 (m, 22H, H3'–H11'), 1.45–1.53 (m, 2H, H2'), 1.76–

1.97 (m, 3H, H4 and H8), 2.03–2.21 (m, 4H, H1', H5), 3.08–3.13 (m, 1H, H2 *trans*), 3.32–3.45 (m, 4H, H6 and H7), 3.73–3.77 (m, 1H, H2 *cis*), 3.85–3.90 (m, 2H, N^+CH_2 ally), 4.12–4.16 (m, 1H, H3), 5.55–5.65 (m, 2H, H3 ally), 5.95–6.08 (m, 1H, H2 ally), 8.29 (d, *J* = 5.97 Hz, 1H, CONH).

^{13}C NMR (DMSO-d_6) δ/ppm: 14.43 (C14'), 18.72, 22.58, 22.68, 25.36, 25.66, 29.02–29.56, 31.75, 34.80, 35.55 (C1'–C13', C5, C8), 24.97 (C4), 44.90 (C3), 53.77 (C6), 53.98 (C7), 59.91 (C2), 65.61 (N^+CH_2 ally), 126.11 (C2 ally), 127.78 (C3 ally), 173.08 (C=O).

N-*allyl-3-hexadecylamidoquinuclidinium bromide* (**QC$_{16}$-Ally**) ^{1}H NMR (DMSO-d_6) δ/ppm: 0.87 (t, *J* = 6.85 Hz, 3H, H16'), 1.21–1.30 (m, 26H, H3'–H11'), 1.45–1.52 (m, 2H, H2'), 1.77–1.96 (m, 3H, H4 and H8), 2.05–2.20 (m, 4H, H1', H5), 3.07–3.12 (m, 1H, H2 *trans*), 3.31–3.44 (m, 4H, H6 and H7), 3.73–3.78 (m, 1H, H2 *cis*), 3.87–3.90 (m, 2H, N^+CH_2 ally), 4.11–4.15 (m, 1H, H3), 5.55–5.64 (m, 2H, H3 ally), 5.98–6.08 (m, 1H, H2 ally), 8.27 (d, *J* = 5.98 Hz, 1H, CONH).

^{13}C NMR (DMSO-d_6) δ/ppm: 14.44 (C16'), 18.71, 22.57, 22.70, 25.35, 25.66, 28.99–29.50, 31.74, 34.77, 35.56 (C1'–C15', C5, C8), 24.96 (C4), 44.90 (C3), 53.77 (C6), 53.97 (C7), 59.90 (C2), 65.63 (N^+CH_2 ally), 126.12 (C2 ally), 127.77 (C3 ally), 173.09 (C=O).

N-dodecyl-3-acetamidoqinuclidinium bromide (**QAc-C$_{12}$**): ^{1}H NMR (DMSO-d_6) δ/ppm: 0.86 (t, *J* = 6.86 Hz, 3H, H12'), 1.20–1.37 (m, 18H, H3'–H11'), 1.56–1.67 (m, 2H, H2'), 1.80–2.15 (m, 4H, H5 i H8), 2.08 (s, 3H, $C\underline{H}_3C$=O), 2.25–2.30 (m, 1H, H4), 2.95–2.98 (m, 2H, H1'), 3.30–3.55 (m, 5H, H6, H7 i H2 *cis*), 3.80–3.95 (m, 1H, H2 *trans*), 4.95–4.98 (m, 1H, H3), 8.32 (d, *J* = 5.98 Hz, 1H, CONH).

^{13}C NMR (DMSO-d_6) δ/ppm: 13.35 (C12'), 18.78, 21.49, 21.68, 27.92, 28.09, 28.19, 28.32, 28.39, 28.41, 29.32–29.56, 30.70 (C2'–C11', C5, C8), 20.85 ($\underline{C}H_3C$=O), 23.15 (C4), 50.86 (C3), 55.05 (C6), 55.65 (C7), 61.62 (C2), 67.36 (C1'), 166.34 (C=O).

N-tetradecyl-3-acetamidoqinuclidinium bromide (**QAc-C$_{14}$**): ^{1}H NMR (DMSO-d_6) δ/ppm: 0.85 (t, *J* = 6.87 Hz, 3H, H14'), 1.21–1.36 (m, 22H, H3'–H13'), 1.56–1.66 (m, 2H, H2'), 1.79–2.14 (m, 4H, H5 i H8), 2.07 (s, 3H, $C\underline{H}_3C$=O), 2.24–2.31 (m, 1H, H4), 2.96–2.99 (m, 2H, H1'), 3.30–3.55 (m, 5H, H6, H7 i H2 *cis*), 3.80–3.95 (m, 1H, H2 *trans*), 4.95–4.98 (m, 1H, H3), 8.32 (d, *J* = 5.99 Hz, 1H, CONH).

^{13}C NMR (DMSO-d_6) δ/ppm: 13.36 (C14'), 18.77, 21.49, 21.68, 27.91, 28.10, 28.17, 28.33, 28.40, 28.41, 29.32–29.56, 30.69 (C2'–C13', C5, C8), 20.86 ($\underline{C}H_3C=O$), 23.17 (C4), 50.84 (C3), 55.07 (C6), 55.65 (C7), 61.62 (C2), 67.36 (C1'), 166.34 (C=O).

N-hexadecyl-3-acetamidoqinuclidinium bromide (**QAc-C16**): ^{1}H NMR (DMSO-d_6) δ/ppm: 0.85 (t, *J* = 6.88 Hz, 3H, H16'), 1.20–1.37 (m, 26H, H3'–H15'), 1.56–1.67 (m, 2H, H2'), 1.80–2.15 (m, 4H, H5 i H8), 2.08 (s, 3H, $C\underline{H}_3C=O$), 2.25–2.30 (m, 1H, H4), 2.98–3.00 (m, 2H, H1'), 3.30–3.55 (m, 5H, H6, H7 i H2 *cis*), 3.80–3.95 (m, 1H, H2 *trans*), 4.95–4.98 (m, 1H, H3), 8.32 (d, *J* = 5.98 Hz, 1H, CONH).
^{13}C NMR (DMSO-d_6) δ/ppm: 13.36 (C16'), 18.78, 21.49, 21.68, 27.91, 28.10, 28.17, 28.33, 28.40, 28.41, 29.32–29.56, 30.69 (C2'–C15', C5, C8), 20.86 ($\underline{C}H_3C=O$), 23.17 (C4), 50.84 (C3), 55.07 (C6), 55.65 (C7), 61.62 (C2), 67.36 (C1'), 166.34 (C=O).

N-dodecyl-3-benzamidoqinuclidinium bromide (**QBn-C12**): ^{1}H NMR (DMSO-d_6) δ/ppm: 0.85 (t, *J*=6.98 Hz, 3H, H12'), 1.20–1.37 (m, 18H, H3'–H11'), 1.62–1.73 (m, 2H, H2'), 1.78–1.85 (m, 1H, H5 *trans*), 1.96–2.00 (m, 2H, H8), 2.16–2.28 (m, 2H, H4, H5cis), 3.13–3.18 (m, 2H, H1'), 3.34–3.53 (m, 5H, H6, H7, H2 *trans*), 3.79–3.84 (m, 1H, H2 *cis*), 4.33–4.38 (m, 1H, H3), 7.48–7.51 (m, 2H, H3, H5 bz), 7.56–7.58 (m, 1H, H4 bz), 7.89 (d, J = 7.16 Hz, 2H, H2, H6 bz), 8.63 (d, J = 5.67 Hz, 1H, CONH).
^{13}C NMR (DMSO-d_6) δ/ppm: 13.36 (C12'), 17.81, 21.49, 21.68, 27.91, 28.10, 28.17, 28.33, 28.40, 28.41, 30.69 (C2'–C11'), 20.78 (C7), 23.65 (C4), 25.31(C5), 44.90 (C3), 52.95, 52.41 (C6, C8), 58.00 (C1'), 62.68 (C2), 126.91 (C2, C6 bz), 127.67 (C3, C5 bz), 130.96 (C4 bz), 133.29 (C1 bz), 166.35 (C=O).

N-tetradecyl-3-benzamidoqinuclidinium bromide (**QBn-C14**): ^{1}H NMR (DMSO-d_6) δ/ppm: 0.85 (t, *J* = 6.98 Hz, 3H, H14'), 1.19–1.35 (m, 22H, H3'–H13'), 1.60–1.73 (m, 2H, H2'), 1.79–1.85 (m, 1H, H5 *trans*), 1.96–2.01 (m, 2H, H8), 2.16–2.26 (m, 2H, H4, H5 *cis*), 3.13–3.18 (m, 2H, H1'), 3.34–3.55 (m, 5H, H6, H7, H2 *trans*), 3.80–3.85 (m, 1H, H2 *cis*), 4.33–4.39 (m, 1H, H3), 7.47–7.52 (m, 2H, H3, H5 bz), 7.56–7.59 (m, 1H, H4 bz), 7.89 (d, J = 7.16 Hz, 2H, H2, H6 bz), 8.64 (d, *J* = 5.67 Hz, 1H, CONH).
^{13}C NMR (DMSO-d_6) δ/ppm: 13.36 (C14'), 17.82, 21.50, 21.69, 27.93, 28.12, 28.19, 28.32, 28.35, 28.40, 28.45, 28.46, 30.70 (C2'–C13'), 20.80 (C7), 23.68 (C4), 25.32(C5), 44.92 (C3), 52.78, 52.95 (C6, C8), 57.92 (C1'), 62.68 (C2), 126.94 (C2, C6 bz), 127.68 (C3, C5 bz), 130.96 (C4 bz), 133.27 (C1 bz), 166.33 (C=O).

N-hexadecyl-3-benzamidoqinuclidinium bromide (**QBn-C16**): ^{1}H NMR (DMSO-d_6) δ/ppm: 0.85 (t, *J* = 6.98, 3H, H16'), 1.20–1.33 (m, 26H, H3'–H15'), 1.62–1.71 (m, 2H, H2'), 1.78–1.85 (m, 1H, H5 *trans*), 1.97–2.00 (m, 2H, H8), 2.16–2.28 (m, 2H, H4, H5 *cis*), 3.14–3.19 (m, 2H, H1'), 3.35–3.54 (m, 5H, H6, H7, H2 *trans*), 3.79–3.84 (m, 1H, H2 *cis*), 4.34–4.39 (m, 1H, H3), 7.48–7.51 (m, 2H, H3, H5 bz), 7.56–7.58 (m, 1H, H4 bz), 7.89 (d, J = 7.16Hz, 2H, H2, H6 bz), 8.64 (d, J = 5.67, 1H, CONH).

^{13}C NMR (DMSO-d_6) δ/ppm: 13.36 (C16'), 17.83, 21.50, 21.70, 27.94, 28.12, 28.20, 28.32, 28.36, 28.42, 28.43, 28.47, 30.70, 31.65, 34.59 (C2'–C15'), 20.81 (C7), 23.68 (C4), 25.33(C5), 44.92 (C3), 52.79, 52.96 (C6, C8), 57.94 (C1'), 62.69 (C2), 126.94 (C2, C6 bz), 127.67 (C3, C5 bz), 130.96 (C4 bz), 133.28 (C1 bz), 166.33 (C=O).

6.2. Supplementary Material S2

Table S1. Minimal inhibitory concentration (MIC/ µM) of 3-aminoquinuclidine and 3-amidoquinuclidine QAC precursors.

**Species**	**Strain origin**	**Minimum inhibitory concentration (MIC)/µM**					
**Gram-positive**		**QNH_2**	**QC_{12}**	**QC_{14}**	**QC_{16}**	**QAc**	**QBn**
Staphylococcus aureus	ATCC 25923	>100	>100	**60**	**62.5**	>100	>100
Staphylococcus aureus	Clinical/MRSA	>100	>100	**50**	250	>100	>100
Staphylococcus aureus	ATCC 33591	>100	100	**50**	100	>100	>100
Bacillus cereus	ATCC 14579	>100	>100	**25**	>100	>100	>100
Listeria monocytogenes	ATCC 7644	>100	100	**25**	125	>100	>100
Enterococcus faecalis	ATCC 29212	>100	>100	**17**	>100	>100	>100
**Gram-negative**							
Escherichia coli	ATCC 25922	>100	>100	50	>100	>100	>100
Salmonella enterica	food isolate	>100	>100	100	>100	>100	>100
Pseudomonas aeruginosa	ATCC 27853	>100	>100	>100	>100	>100	>100

6.3. Supplementary Material S3

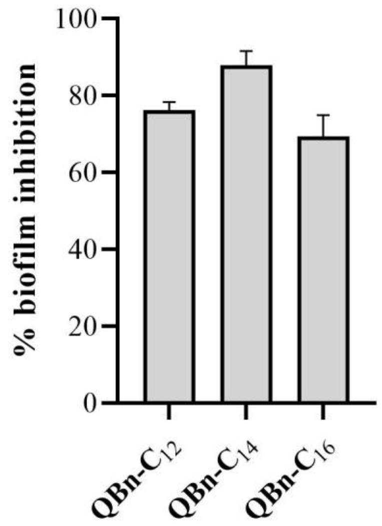

Figure S1. *Staphylococcus aureus* ATCC 25923 biofilm inhibition of the series **3** candidates at the compound concentration of 50 µg mL^{-1}.
